# Privacy-preserving federated prediction of health outcomes using multi-center survey data

**DOI:** 10.1186/s12874-026-02785-5

**Published:** 2026-02-04

**Authors:** Supratim Das, Mahdie Rafiei, Paula T. Kammer, Søren T. Skou, Dorte T. Grønne, Ewa M. Roos, André Hajek, Hans-Helmut König, Md Shihab Ullah, Niklas Probul, Jan Baumbach, Linda Baumbach

**Affiliations:** 1 Department of Health Economics and Health Services Research, University Medical Center Hamburg-Eppendorf, Martinistraße 52, Hamburg, 20246 Germany; 2 Institute for Computational Systems Biology, University of Hamburg, Albert-Einstein-Ring 8-10, Hamburg, 22761 Germany; 3 Department of Sports Science and Clinical Biomechanics, Center for Muscle and Joint Health, University of Southern Denmark, Campusvej 55, Odense, 5230 Denmark; 4 Department of Physiotherapy and Occupational Therapy, The Research and Implementation Unit PROgrez, Naestved-Slagelse-Ringsted Hospitals, Faelledvej 2C, Slagelse, 4200 Denmark; 5 Department of Mathematics and Computer Science, Computational Biomedicine Lab, University of Southern Denmark, Odense, Denmark

**Keywords:** Federated learning, Survey data, Osteoarthritis, Federated linear regression, Federated logistic regression, Federated random forest

## Abstract

**Background:**

Patient-reported survey data are used to train prognostic models aimed at improving healthcare. However, such data are typically available multi-centric and, for privacy reasons, cannot easily be centralized in one data repository. Models trained locally are less accurate, robust, and generalizable. We aim to investigate the applicability of privacy-preserving federated machine learning techniques for prognostic model building on health survey data, where local data never leaves the legally safe harbors of the medical centers.

**Methods:**

We used centralized, local, and federated learning techniques on two healthcare datasets (*GLA: D*^®^data from the five health regions of Denmark and international SHARE data of 27 countries) to predict two different health outcomes. We compared linear regression, random forest regression, and random forest classification models trained on local data with those trained on the entire data in a centralized and in a federated fashion.

**Results:**

In GLA: D^®^ data, federated linear regression (R^*2*^ 0.34, RMSE 18.2) and federated random forest regression (R^*2*^ 0.34, RMSE 18.3) models outperform their local counterparts (i.e., R^*2*^ 0.32, RMSE 18.6, R^*2*^ 0.30, RMSE 18.8) with statistical significance. We also found that centralized models (R^*2*^ 0.34, RMSE 18.2, R^*2*^ 0.32, RMSE 18.5, respectively) did not perform significantly better than the federated models. In SHARE, the federated model (AC 0.78, AUROC: 0.71) and centralized model (AC 0.84, AUROC: 0.66) perform significantly better than the local models (AC: 0.74, AUROC: 0.69).

**Conclusion:**

Federated learning enables the training of prognostic models from multi-center surveys without compromising privacy and with only minimal or no compromise regarding model performance.

**Supplementary Information:**

The online version contains supplementary material available at 10.1186/s12874-026-02785-5.

## Background

Patient-reported survey data have emerged as a valuable tool for assessing medical conditions in clinical settings, serving as a gold standard in various fields [1–4]. Patient-reported survey data and objective functional tests have been instrumental in developing prognostic models using machine learning technique [5–7]. Beyond its applications in personalized medicine, patient-reported survey data is also utilized in health economics research.

Health survey data is collected and stored across local hospitals and practices and sometimes across regions, states, or countries. Local data from a single silo is, by nature, smaller and hence leads to reduced accuracy and generalizability of prognostic statistical models as compared to models trained on larger data sets integrated from several clinics, regions, or countries [8, 9]. The prime reasons for data decentralization are data security and privacy policies. Healthcare data exchange across country borders is particularly challenging [10]. Hence, real-world health survey data is scarce and rarely available for machine learning. In recent years, the idea of a decentralized electronic health record repository network across Europe has been suggested (but not yet addressed or implemented) in the framework of the European Union’s proposal of a European Health Data Space (EHDS) [11].

### Privacy regulations

Different privacy regulations around the world [12–16] effectively prohibit companies and researchers from directly sharing personal patient data. Despite patient ID anonymization attempts [17], recent studies demonstrate how various reidentification techniques may compromise patient privacy [18, 19]. In a recent paper from 2022, Jill Evans et al. have pointed out the challenges in directly sharing osteoarthritis data; besides privacy, they mentioned logistical and structural barriers, such as the limitation of resources for data governance [20].

### Consequences of scattered data silos in medicine

**Fig. 1:**
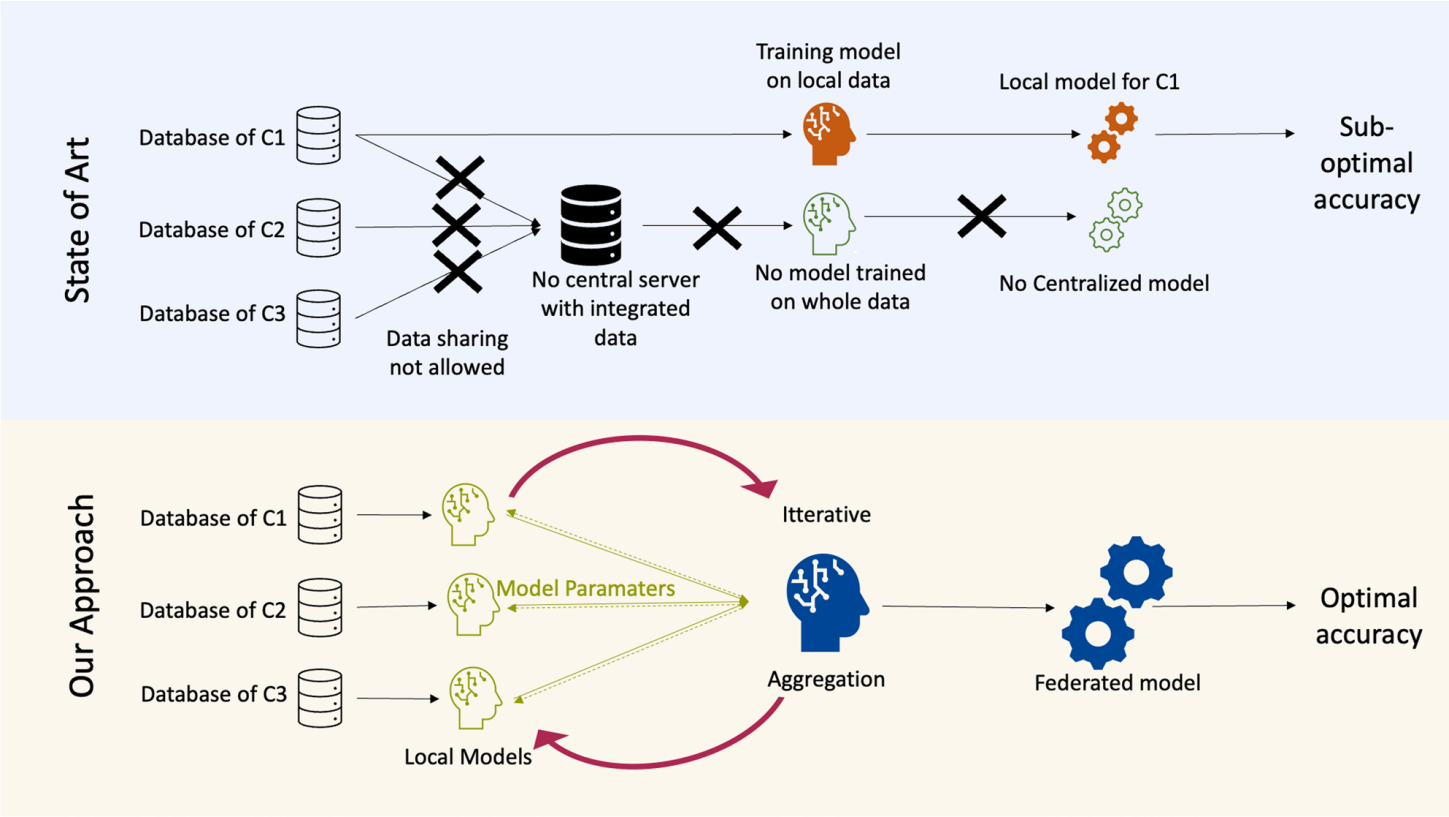
Comparison of traditional machine learning and federated learning: On the top half in the azure background, we represent the current state of the art of model predictions. In traditional machine learning, all data from data centers c1, c2, and c3 must be integrated into one server. Due to privacy issues and other logistic issues, integrating data from all local data centers to one central data site may not be possible. Hence, these traditional machine learning studies are often limited to training individual local models on limited data. These models have suboptimal performance as they fail to generalize in a global scenario. In the bottom part, in the ivory background, we depict our proposed model of federated learning. Here, the raw data never leaves their local servers c1, c2, and c3, and the necessity of data integration in one data center is mitigated. Each data center trains a local model; only model-specific parameters are communicated during training. As the federated machine learning model is trained using insights from all datasets, it can generalize better and potentially give better performance than a local model

As a result, researchers are often constrained to train prognostic models solely based on local data, leading to suboptimal accuracy and potentially incorrect predictions (see Fig. 1 for an illustration). In particular, as local data, in addition to its smaller size, possesses smaller sample diversity, the resulting prognostic machine learning models suffer from reduced generalizability and lead to increased discrimination of underrepresented minorities. Although a model trained on data from one clinical site might learn to predict with high accuracy for that particular clinic, the model might learn from some clinical site-specific information irrelevant to the disease in general [8, 9]. Employing data from multiple hospitals for model training instead of relying solely on more homogeneous single-clinical data has been demonstrated to reduce model bias. Consequently, it leads to improved performance across the whole target population [8, 21]. Besides this, even in scenarios where data centralization is possible, researchers need to go through many legal procedures and would need to take care of data storage logistics [20]; federated learning cuts through these procedures, making data from several locations easy to use.

### Federated learning

To circumvent legal and ethical data-sharing issues while allowing for the training of robust models on big data, researchers have developed federated learning [22, 23] over the last few years. In federated learning, raw data can stay in the legally safe harbors of the local data centers. Instead, one trains a joint model using inference from all data distributed across the different hospitals (see Fig. 1 for an illustration of the concept). For instance, federated learning has been used in computer vision [24] and natural language processing [25]. The efficacy of federated learning has been studied on image data [24], multi-omics data [26, 27], and other biomedically relevant data types [23, 28, 29]. The applicability of a federated model in such data is often determined based on its ability to outperform local models [30, 31] while maintaining a close resemblance to centralized models [30–32]. However, its potential and effectiveness for decentralized profiling of patient-reported survey data have been unexplored hitherto. The nature of survey data differs significantly from data obtained through genomic analyses, standard biochemical assays, or radiographic imaging. Unlike the continuous data typically encountered in these domains, survey data is often in the form of ordinal or nominal data. While ordinal data may be approximated as continuous under certain circumstances, it deviates from the characteristics of true continuous data [33].

Here, we aim to fill this gap by investigating the applicability of federated learning to patient-reported survey data. First, we predicted the development of self-reported pain in patients with osteoarthritis using data from all five regions of Denmark, based on previous work utilizing linear and random forest regression models [5]. Second, we use random forest classification models to predict physical inactivity using data from 27 European countries.

We anticipate that, despite the distinct nature of our dataset, federated learning, consistent with previous studies, exhibits superior accuracy compared to local models across all scenarios [34]. Similarly, based on findings from existing studies, we anticipate comparable performance to the centralized counterparts [34, 35], for the federated linear regression, random forest regression, and random forest classification.

## Method

### Data and preprocessing

For the first task, we utilized data from the*GLA: D*^®^(Good Life with osteoArthritis in Denmark) registry [36, 37]. National GLA: D^®^ databases exist in Australia, Austria, Canada, China, Germany, Ireland, Netherlands, New Zealand, and Switzerland [36–39]. The*GLA: D*^®^initiative consists of three parts: a certification course for physiotherapists, a standardized and supervised patient education and an exercise therapy program, and a registry with data from before, immediately after, and 12 months after program initiation [36–39]. *GLA: D*^®^aims to facilitate the implementation of recommended first-line pain management (exercise therapy and patient education) for patients with hip and knee osteoarthritis in clinical practice [37].

We utilized Danish data from the beginning of 2013 to the end of 2018. We used the same preprocessing described in a paper on individualized predictions of changes in knee pain for patients with knee osteoarthritis using the same data source [5], which includes a selection of the same 51 variables and dropping of patient data samples with missing values. Thus, we only included complete cases. To simulate a realistic federated learning scenario, we then split the data according to the five Danish administrative health care regions (North Denmark, Central Denmark, Southern Denmark, Zealand region, and the Capital Region of Denmark) using the geographical location where the (*n* = 274) *GLA: D*^®^ clinics are located [39]. For this, we call this task a national scenario.

We trained statistical models to predict changes in knee pain intensity over the period of approximately three months, from before to after the *GLA: D*^®^ program. We utilized the same 51 variables as Baumbach et al.[5] used. A detailed description of these 51 variables as selected based on clinical reasoning by the authors of the aforementioned publication, providing information about the patient’s demographics and health status, is provided in supplementary table B.1. Knee/hip pain intensity was measured on a VAS scale of 0 to 100 mm, best to worst. Hence, the possible values for pain change are between − 100 and 100. The script used for preprocessing can be accessed in our GitHub repository [40].

For the second task, we used the data from wave 8 (released in 2022) of the Survey of Health, Ageing, and Retirement in Europe (SHARE) database [41, 42] to predict physical inactivity. We chose this dataset because of the high heterogeneity in physical activity across 27 countries [42]. Hence, we expected this to impose greater challenges on the federated model. We calculated our outcome variable physical inactivity (yes/no) from two questions as suggested by Gomes et al.[43]. Afterwards, we selected 30 relevant variables, including information on mental health (such as EURO-D measure of depressive symptom [44, 45]), cognitive abilities, movement limitations, self-perceived health, which are shown to be relevant factors in the literature [43] [see supplementary Table B.3.] To simulate federated learning across multiple countries, we split the data into 27 countries. A full list of these variables and data preprocessing codes can be found in our GitHub repository [40].

In either scenario, we only harmonize the data in terms of selecting the same variables. However, we do not artificially introduce data bias or balance existing data bias, as in real life, one would expect such data bias, and an ideal federated model should be able to handle that.

#### Local vs. centralized vs. federated machine learning

**Fig. 2:**
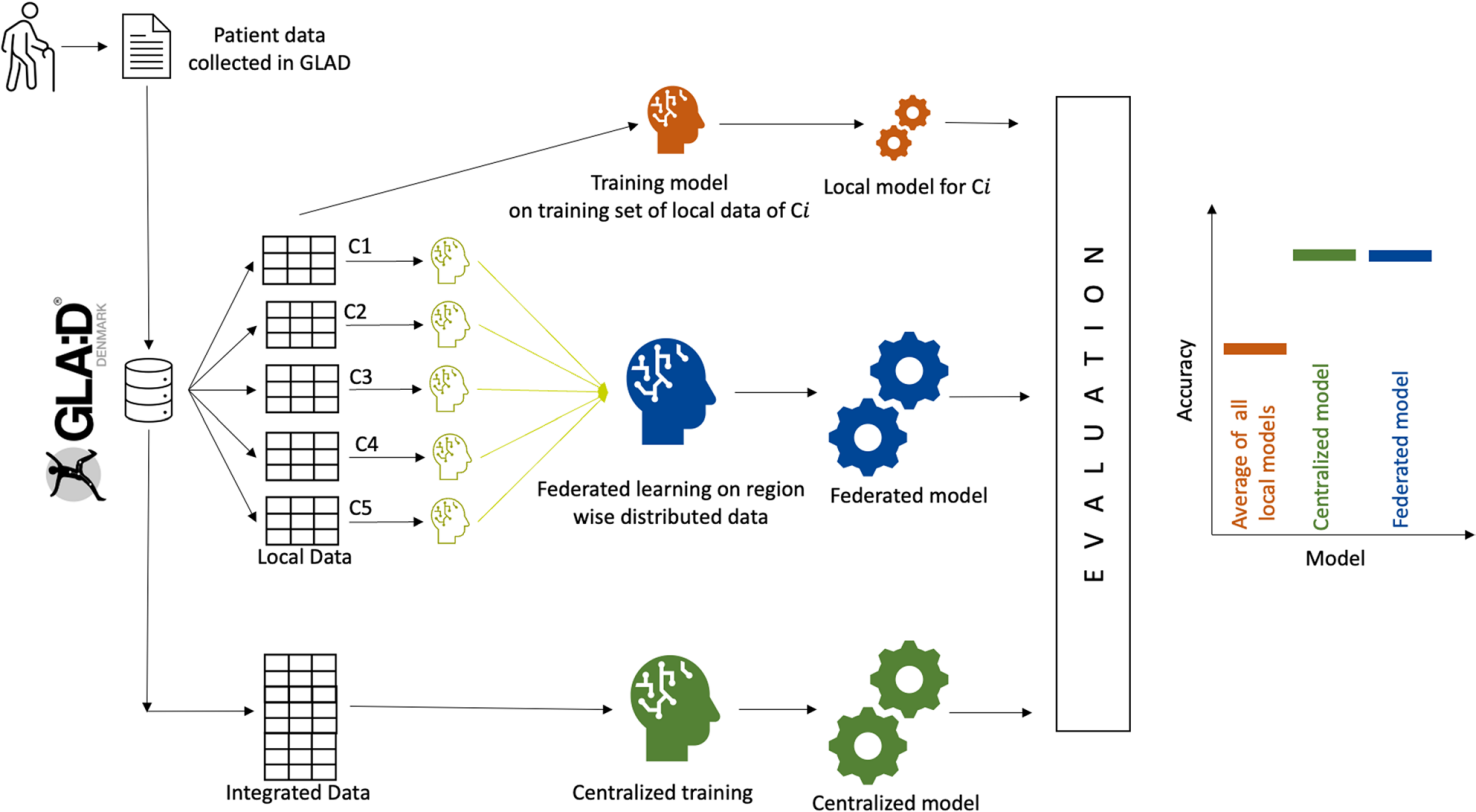
Representation of the evaluation scheme for Federated Learning, exemplarily depicted for five regions of *GLA:D*® Denmark’s data: To compare with a centralized scenario, we simulate a scenario of distributed and centralized data centers using Danish *GLA:D*® data. We trained five local models with each region’s local data, one federated model with distributed data, and one centralized model with integrated data. We evaluated this protocol in VAS pain prediction from 51 variables using linear regression and random forest regression as our machine-learning models of choice. We compared the average of 5 local models and a centralized scenario with the federated model for both machine learning algorithms (linear regression and random forest). A similar scheme was executed with the SHARE data but for 27 countries using random forest classification

We trained the machine learning algorithms for both tasks in three different scenarios:

- A. Local models: For task one, five separate models were trained in isolation on the local *GLA: D*^®^ data of the five regions of Denmark. Similarly, for task two, 27 separate models were trained in isolation on local data from the 27 countries participating in SHARE (wave-8). In task two for the classification task, before training the model for each country, the data was balanced (same number of positive and negative samples) by supersampling (sampling with replacement from the smaller class).
- B. Centralized model: For task one, local *GLA: D*^®^ data of the five training sets were merged and used together to train one centralized model while having access to the full data. For task two, country-wise supersampled balanced training data sets of SHARE data were combined to create the international, centralized training set.
- C. Federated model: For both tasks, we trained the federated model using the same data used for local training, but while keeping the regional/national sets separated (see Fig. 2).

Finally, we trained linear regression, random forest regression, and random forest classification models for each of the three scenarios and evaluated their performances.

### Training and test data generation

For both tasks, we performed 5-fold cross-validation, which gives us an 80% sample for training and a 20% sample for testing the machine learning models in each fold (see Supplementary Figure A.2). For task one, for each fold, during training, the federated and local models utilized the train split of their local data, while for centralized training, the same training splits are concatenated to form a centralized training set. In each fold, we used the global test set to evaluate the performance of local, centralized, and federated models. We tested all scenarios on the same global test set (see Supplementary Figure A.2).

Similarly, for task two, we split the data according to 27 countries, and in each country, we made a training-test split in each fold of cross-validation. For the training split, we balanced the dataset by supersampling. For evaluation, we first merged the local test sets into centralized ones; then, we created 4 subsampled balanced subsets where the sample of the larger class is mutually exclusive in the 4 subsets. We do this for fair evaluation on a balanced test set.

### Machine learning methods

#### Scenario 1: local models

For the local models, we used linear regression, random forest regression, and random forest classification models trained on a training split of the respective local data of each of the regions (for task one) or countries (for task two). Thus, we have five separate models for each algorithm in each fold. For all local models, we utilized the Sci-Kit Learn library [46] in Python.

#### Scenario 2: centralised model

For the centralized models, we used the same Sci-Kit Learn library in Python to train linear regression, random forest regression, and random forest classification models. Here, for every machine learning model, only one model is trained in each fold, which utilizes the integrated training data (see Supplementary Figure A.2).

#### Scenario 3: federated model

In federated learning, instead of sharing raw data, each data center or clinic, also referred to as “client,” locally performs computations on their local data and only shares model parameters with an aggregator [3, 9, 21–23]. The aggregator then combines all those parameters and sends them back to each client. Now, each client updates their machine learning model using these values. In iterative training schemes (like random forest classification, for instance), these two steps are iterated until the model converges. See Supplementary Figure A.1 for an illustration. Federated learning, in brief, works as follows:

1. Initialization: All clients are initialized with the same model architecture; this might be created from a local configuration file or received from the coordinator, which can be an external or one of the participating clients. The coordinator can be the same as the aggregator.
2. Local training: At this stage, each local client trains their local model using local data. For an iterative model, these local models are trained for *s* local gradient descent steps before sending parameters for the communication step.
3. Communication: In this step, each client sends local parameters or local gradients to the aggregator.
4. Global aggregation: The aggregator combines all the received local parameters from the clients and updates the shared model. This can be performed using simple weighted averaging.
5. Convergence: Steps 2–4 are repeated until preset convergence criteria are met. The federated random forest classifier is implemented as an iterative model.
6. Model evaluation: After training termination, each client saves the model, performs a prediction on the local test set, and outputs local results for evaluation.

For federated learning, we used the feature cloud platform [34], which provides users with an interactive user interface to run commonly used machine learning algorithms in a federated fashion.

We implemented federated linear regression with secure multi-party communication (SMPC) by sharing *X*^⊤^◦ *X* and *X*^⊤^◦ *Y*[47], where *X* is an *n*,* m* matrix of *n* samples and *m* features, and *Y* is the target variable vector. For the federated random forest regression and classification models, local random forest regressor models were first trained and shared with ε-δ differential privacy (DP) where ε = 1, δ = 1*$\:1{0}^{-5}$.Then these shared estimators of random forest or decision trees were gathered to create a new random forest model in the aggregation stage [34]. Once the models were trained, we performed SHapley Additive exPlanations (SHAP) analysis on the federated and centralised models and plotted the mean of absolute of SHAP values of each feature on the test set. Detailed information on the utilized FeatureCloud app versions and the applied configuration files can be found in our GitHub repository [40].

#### Evaluation

For all the models, we first evaluated their performance using 5-fold cross-validation. In all three scenarios (local, centralized, and federated), we calculated R-squared and root mean squared error (RMSE) on the global test data to evaluate linear regression and random forest regression. For the Random forest classifiers in each fold of cross-validation, we tested 4 different subsampled unbiased centralized test sets for fairness. In those 4 test sets, the negative samples were mutually exclusive. For evaluation, we used both accuracy and AUROC.

We performed single-sided Welch t-test tests to check our hypotheses on whether centralized and federated models outperform the local, and whether the centralized analyses perform better than federated ones. Python scripts for evaluation and statistical tests are available through our GitHub repository [40].

#### Criteria for the applicability of federated learning

Being among the first to apply federated learning to survey-based data, we used literature from computer vision and other biomedical data to define when federated learning would be applicable to survey data. In computer vision tasks, comparisons to local models are often omitted, and the threshold for determining federated algorithm performance relative to centralized models remains unspecified [48–50]. In federated learning literature, only an improvement over other federated algorithms is reported [51, 52]; the statistical significance tests are often left out. The precise cutoff for the extent of similarity to centralized accuracy remains undefined in the literature and depends on the specific application and the trade-off between privacy and accuracy. In light of these findings, we adopt the following two criteria: if the federated model’s accuracy surpasses that of local models and aligns closely with centralized model performance, we deem it applicable. Thus, a necessary criterion to deem federated learning applicable is that the federated models perform statistically significantly better than local models. However, if centralized models do not outperform federated models, it is a sufficient criterion.

### Additional analyses

For the tasks based on the GLA: D^®^ data, we performed some additional analysis to test if the regions are statistically significantly different from each other and how they perform in the data scarcity scenario.

#### Subsampling experiment

To investigate the efficacy of federated learning in scenarios where each client possesses limited data, we replicated our experiments based on the GLA: D^®^ data with reduced training data. The training set size was progressively reduced to 75%, 50%, 25%, and 10% of its original size, while the central test set remained unchanged. We compared the performance of linear regression and random forest models trained locally and centrally using federated learning under these reduced data conditions. For the 25% subsampling, we employed a five-fold cross-validation procedure. We performed the same evaluation and statistical tests to produce results comparable to those obtained with the entire training set.

#### Data heterogeneity

We also tested the heterogeneity of all variables across all the regions, as data heterogeneity might specifically affect the performance of federated models. For each of the 51 independent variables and the target variable, we assessed every pair of regions to determine whether their data originated from distinct distributions. This analysis involved conducting ANOVA, followed by post-hoc comparisons using the Scheffé method. To evaluate the extent of the effect size of the difference in distribution, we used Cohen’s D.

## Results

After preprocessing the *GLA: D*^®^data, we included 9,648 patients. The number of samples per region is given in Table 1.

**Table 1:** Distribution of patients in *GLA: D*^®^registry across five different regions of Denmark

Region name	Region ID	number of patients
Capital Region of Denmark	1	2415
Region Zealand	2	1550
Region of Southern Denmark	3	2530
Central Denmark Region	4	2220
North Denmark Region	5	933
total	.	9648

For the SHARE dataset, we included 46,570 patients from 27 countries. The number of samples per region varies between 2933 and 509 participants; details are provided in supplementary Table 2.

### Performance evaluation

All results for comparisons of federated models with centralized and local models are given below in Table 2. The p-values for the statistical tests are given in Table 3.

**Table 2:** Comparison of federated learning with centralized and local models when trained on the full *GLA: D*^® ^dataset and wave 8 of the SHARE dataset

data/scenario	Model	Metric	Average of Locals	Centralized	Federated	Relative Improvement of federated over local
*GLA: D*^®^, National	Linear Regression	r-square	0.32	0.34	0.35	9.37%
*GLA: D*^®^, National	Linear Regression	RMSE	18.6	18.2	18.2	2.15%
*GLA: D*^®^, National	Random Forest Regression	r-square	0.30	0.32	0.34	13.33%
*GLA: D*^®^, National	Random Forest Regression	RMSE	18.8	18.5	18.3	2.65%
SHARE, International	Random Forest Classification	accuracy	0.82	0.85	0.84	2.43%
SHARE, International	Random Forest Classification	AUROC	0.75	0.76	0.79	5.33%
SHARE, International	Random Forest Classification	F1 Score	0.41	0.44	0.45	9.75%
SHARE, International	Random Forest Classification	AUPRC	0.47	0.47	0.54	14.83%

**Table 3:** Statistical tests for comparison of federated learning with centralized and local models when trained on the full ***GLA: D***^®^ dataset and wave 8 of the SHARE dataset. We mark non-statistically significant changes (i.e.,. *p*-values ≥ 0.05) with “n.s.”

data/scenario	Model	Metric	If Centralised is better than Local (*P*-value)	If Federated is better than Local (*P*-value)	If Centralised is better than Federated (*P*-value)
*GLA: D*^®^, National	Linear Regression	r-square	0.0008	0.0009	0.48 (n.s.)
*GLA: D*^®^, National	Linear Regression	RMSE	0.06(n.s.)	0.03	0.63(n.s.)
*GLA: D*^®^, National	Random Forest	r-square	0.012	5.6 $\:\times1{0}^{-6}$	0.99(n.s.)
*GLA: D*^®^, National	Random Forest	RMSE	0.12 (n.s.)	0.004	0.95(n.s.)
SHARE, International	Random Forest Classification	accuracy	2.7 $\:\times1{0}^{-9}$	1.5 $\:\times1{0}^{-11}$	0.005
SHARE, International	Random Forest Classification	AUROC	9.3 $\:\times1{0}^{-8}$	0.0001	0.99
SHARE, International	Random Forest Classification	F1 Score	2.1 $\:\times1{0}^{-6}$	0.001	0.82
SHARE, International	Random Forest Classification	AUPRC	0.77	0.0009	0.99

#### Linear regression

**Fig. 3:**
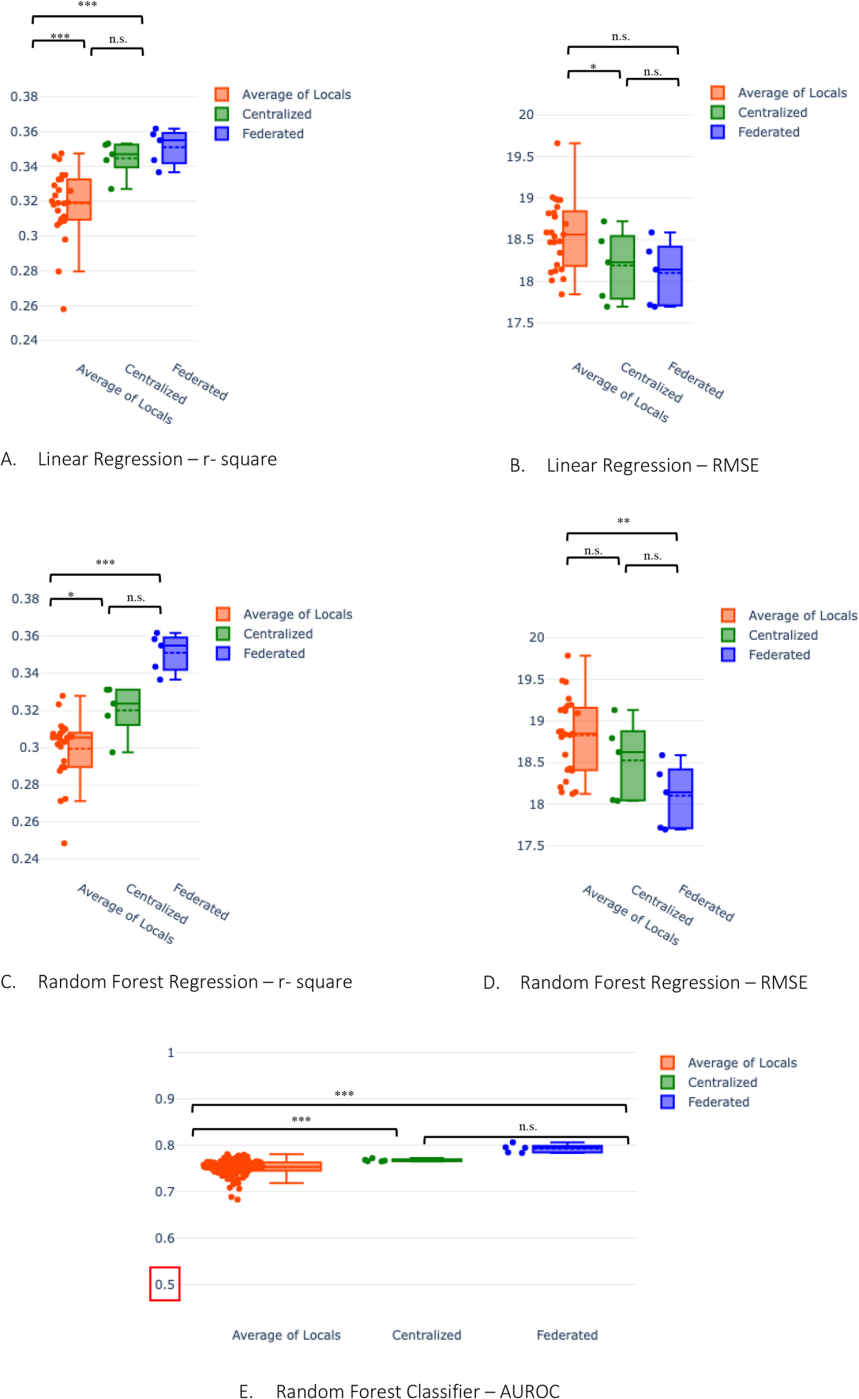
Comparison of federated model with centralized and local models: For both **A.** R-square of linear regression (higher the better) and **B.** RMSE plot of linear regression (lower the better), we see federated and centralized models performing better than the local models. Similarly, for both **C.** R-square of random forest regression (higher the better) and panel **D.** RMSE of random forest regression (lower the better), we see that the federated model outperforms both local and central models. In **E.** For the random forest classifier, we see that the federated model has a higher AUROC than centralized and local. The dotted lines in the box plots represent the mean, while the solid lines represent the median. The stars represent the magnitude of the significance of the tested hypothesis in the following convention: *** : p<0.001, **: p<0.01, * : p<0.05, n.s. : p>0.05. We tested 1. if the centralized model is better than the average of local ones, 2. if the federated model is better than the average of local ones, and 3. if the centralized model is better than the federated model

For linear regression used in task 1, federated learning models have a higher R-squared and lower RMSE than the corresponding average of the local models (R-squared p-val 0.0009, RMSE p-val 0.03). While the federated model achieves R-squared 0.35 and RMSE 18.2, the local models, on average, achieve R-squared 0.32 and RMSE 18.6 (Table 2). Conversely, the centralized model achieves R-squared 0.34 and RMSE 18.2 (Table 2). Hence, the centralized model does not outperform the federated model (R-squared p-val 0.48, RMSE p-val 0.63)(Table 3). Note that the local model trained on data from the North Denmark Region (Region 5) performs the worst among the local models (Fig. 3). We also see the top 5 key predictors according to SHAP analysis remain unchanged in federated from centralized (see Supplementary figure A.4.).

#### Random forest regression

**Fig. 4:**
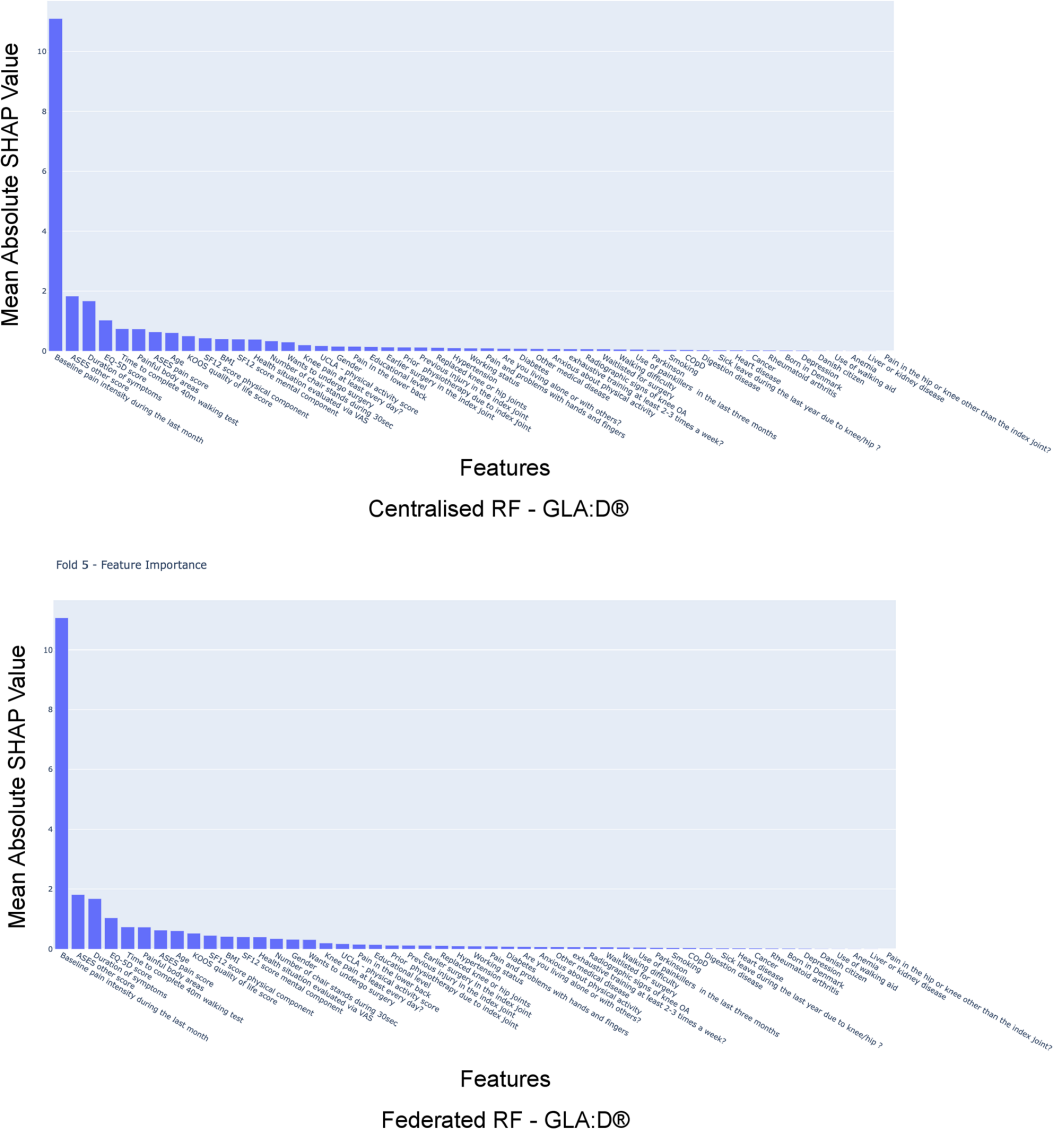
Comparison of SHAP-based feature importance federated Random Forest with centralized Random forest: Mean of absolute of SHAP value of all the features for both centralised and ploted as hhistogram, we see the most 5 features in both are preserved in same order the top predictor of change in knee pain (VASA scale) in 3 months being base level pain in VAS scale

For random forest regression used in task 1, federated learning models have a higher R-squared and lower RMSE than the corresponding average of the local models (R-squared p-val 5.6 $\:*1{0}^{-6}$, RMSE p-val 0.004). While the federated model achieves R-squared 0.34 and RMSE 18.3, the local models, on average, achieve R-squared 0.30 and RMSE 18.8 (Table 2). Conversely, the centralized model achieves R-squared 0.32 and RMSE 18.5 (Table 2). Hence, the centralized model does not outperform the federated model (R-squared p-val 0.99, RMSE p-val 0.95) (Table 3). Rather, the federated model outperforms the centralized model (Fig. 3). We also see that the top 5 key predictors according to SHAP analysis remain unchanged in federated from centralized (Fig. 4).

#### Random forest classification

In random forest classification, used in task 2, the centralized model accuracy slightly outperforms(0.85) the federated model (0.84). Both models outperform the local model (0.82) (Supplementary Figure A.3). However, the federated model achieved a higher AUROC (0.79) than the centralized (0.76) and the average of local models (0.75) (Fig. 3). A higher accuracy but lower AUROC in the centralized model over the federated model suggests that federated random forest classification is more robust and less prone to overfitting. We also see the top 5 key predictors according to SHAP analysis remain unchanged in federated from centralized (see Supplementary figure A.4).

### Supplementary analyses

#### Subsampling experiment

**Fig. 5:**
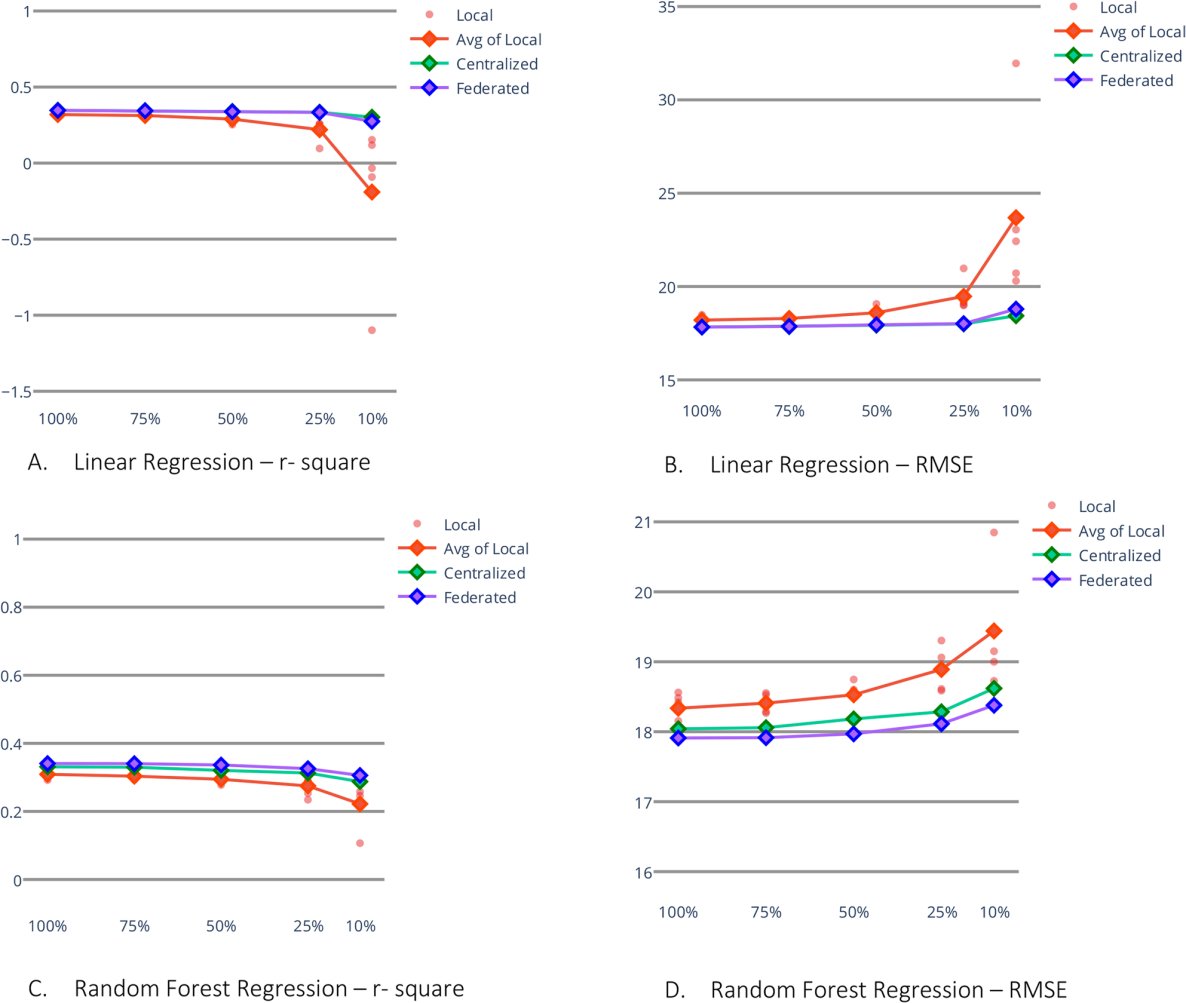
Error rates after training set subsampling experiment in *GLA:D*®: The training data has been subsampled to 75%, 50%, 25%, and 10%, while test data is kept untouched. Linear regression and random forest regression are trained on subsampled *GLA:D*® data to compare federated models with centralized and local models. For both machine learning experiments, although the local model, on average, performs worse than the federated model, the difference between them intensifies with a reduction in the sample size of training data

In the first task, the performance gap between local models and centralized models widens as the training dataset size diminishes (Fig. 5). Still, federated models manage to keep up with their centralized counterparts. In the 25% subsampled training set experiment (range *n* = 88 to *n* = 266), we observe a remarkable 55.3% improvement in R-squared for linear regression when using federated learning compared to the average performance of local models across five-fold cross-validation (Table 4 and Supplementary Figure A.4.). Federated random forest exhibits a substantial 20.9% improvement in R-squared value over its local counterpart. Unlike the experiment in full data in this data-scarce simulation, the statistical test always finds a statistically significant improvement of centralized and federated models over local models. However, there is no significant improvement in the centralized over the federated model (Table 5).

**Table 4:** Comparison of federated learning with centralized and local models when trained on only 25% of the training data in *GLA: D*^®^

Model trained on 25% of the training data	Metric	Average of Locals	Centralized	Federated	Relative Improvement of federated over local
Linear Regression	r-square	0.21	0.31	0.35	55.32%
Linear Regression	RMSE	19.9	18.7	18.5	7.42%
Random Forest Regression	r-square	0.27	0.31	0.32	20.83%
Random Forest Regression	RMSE	19.2	18.6	18.5	3.89%

**Table 5:** Statistical tests for comparison of federated learning with centralized and local models when trained on only 25% of the training data In *GLA: D*^®^. We denote the non-significant* p*-values ≥ 0.05 with n.s. In parentheses

Model	Metric	If Centralised is better than Local (*P*-value)	If Federated is better than Local (*P*-value)	If Centralised is better than Federated (*P*-value)
Linear Regression	r-square	1.23 $\:\times1{0}^{-6}$	1.80 $\:\times1{0}^{-7}$	0.95(n.s.)
Linear Regression	RMSE	0.00067	1.01 $\:\times1{0}^{-5}$	0.75(n.s.)
Random Forest Regression	r-square	0.00045	1.35 $\:\times1{0}^{-8}$	0.98(n.s.)
Random Forest Regression	RMSE	0.021	3.39 $\:\times1{0}^{-3}$	0.73(n.s.)

#### Data heterogeneity

**Fig. 6:**
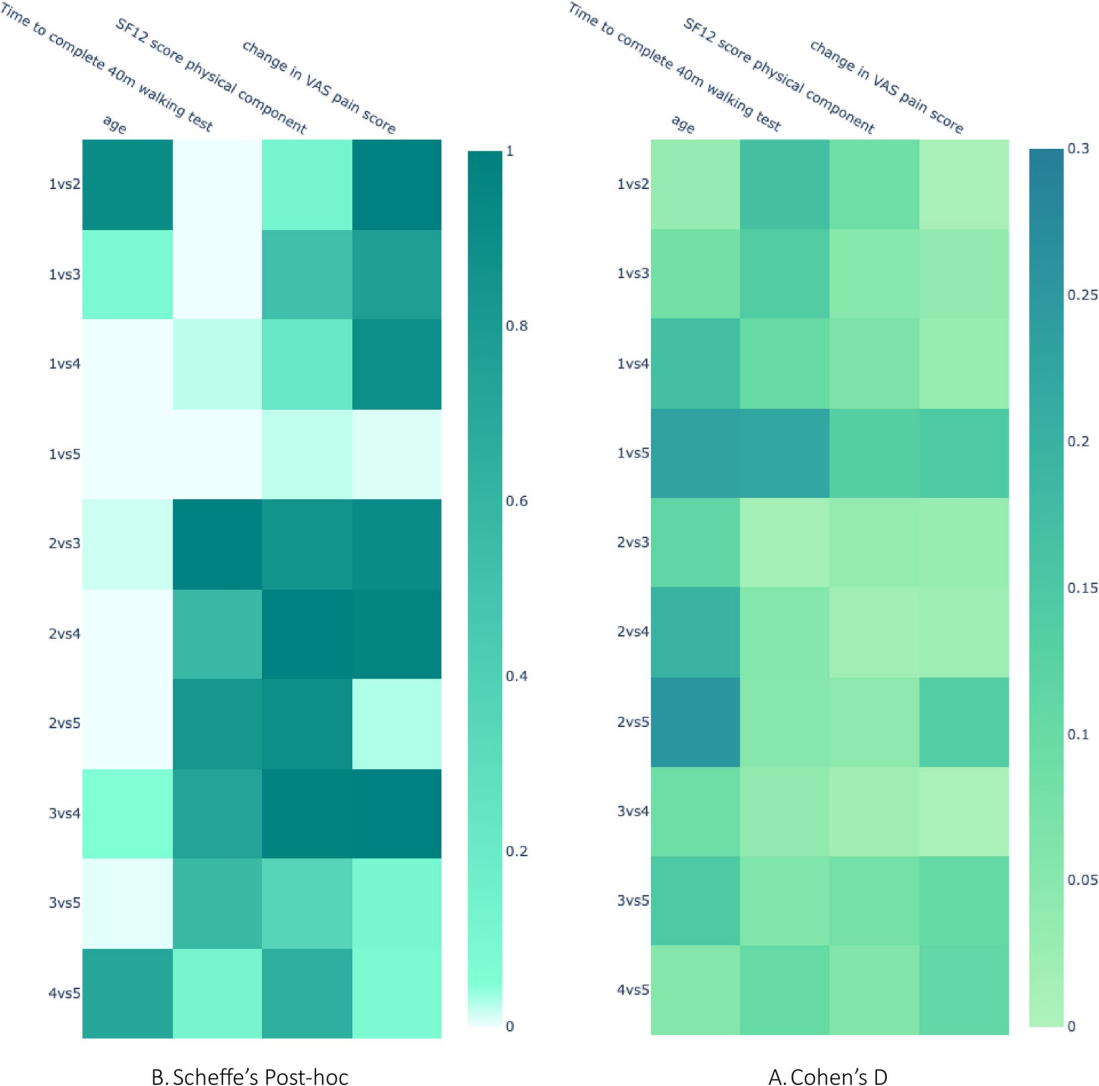
Statistical test to evaluate data heterogeneity of *GLA:D*® across five regions: **A **Post hoc Scheffe test: We used post hoc Scheffe to test pairwise if two regions’ data distributions differ. The comparison of two regions, X and Y, is denoted as “XvsY.” We did this for all 51 variables and the target variable. Here, we plot this for only 3 of the variables: age, a functional test for time to complete 40 min walking test, SF12 score physical component, and our target variable: Change in VAS pain score. The light color suggests statistical significance, while the darker color suggests no significance. **B **Cohen’s D effect size of each statistical test.: the darker the color, the higher the effect size. Although the means of the distribution are close, we find statistically significant differences among regions in some variables, including the target variable. Find detailed post hoc Scheffe and Cohen’s D analysis plots of all variables in the supplementary material

For the national scenario, in Danish *GLA: D*^®^ data, although the means of the distribution of each region per each variable are very close and most groups do not differ statistically significantly, we observed some statistically significant differences (Fig. 6(a), supplementary Figure A.6(a) and supplementary table B.4.) in some of the variables (age, change in VAS pain score). In terms of multiple variables, region five (North Denmark Region) is significantly different (lighter color in Fig. 6(a) and Supplementary Figure A.6(a) for region 1 vs. 5, 2 vs. 5, 3 vs. 5, 4 vs. 5). We also tested the Cohen’s D effect size to determine what population of one group is below the average of the mean of the other group. Our results suggest that although most of them have small effect sizes, a few of them (e.g., age, change in VAS pain score) have larger effect sizes (Fig. 6(b) and Supplementary Figure A.6 (b)). Cohen’s D effect size concurs with the post hoc Scheffe analysis (Fig. 6 and Supplementary Figure A.6).

## Discussion

Following the set criteria for the applicability of federated learning, our findings indicate that federated learning applied to patient-reported survey data outperforms models trained on local data with statistical significance, while the centralized models are comparable to the federated model.

In federated learning, sometimes a compromise in accuracy is acceptable for privacy. For example, a compromise of approximately 5% accuracy in brain tumor prediction from MRI images is considered acceptable in federated convolutional neural networks for privacy preservation [32]. However, our federated models in *GLA: D*^®^ data never underperform the centralized models. In SHARE data, although the federated random forest classifier had lower accuracy than centralized, it has higher AUROC than centralized and, hence, is more robust.

Across all three machine learning models investigated, federated models exhibited higher accuracy compared to their local counterparts, as expected. Federated linear regression achieved performance identical to that of its centralized counterpart (same R-squared and accuracy, respectively). Remarkably, the federated random forest regressor in task 1 (R-squared = 0.34) and federated random forest classifier in task 2 (AUROC = 0.79) even surpassed the performance of the centralized counterparts (0.32, 0.75, respectively), a rare phenomenon that has also been observed in multiple biological data [33]. We speculate that this happens due to the overfitting of the centralized random forest regression model. For task 2, we find that the centralized random-forest classifier model overfits, resulting in higher accuracy (0.84) and lower AUROC (0.66) than the federated model (AC 0.78, AUROC 0.71). This suggests that the federated random forest classification model, although it has slightly lower accuracy, is more robust.

We also sometimes see a few local models having very good performance against centralized test sets, while most other local models underperform. However, in real life, it would not be possible to find out the best local model against centralised test sets. In our simulations, we had the “luxury” of comparing different local models against a centralized ground truth by experimental design. In a real-world scenario, this would be impossible.

Our experiment in international data suggests the applicability of federated learning algorithms, even when each country has a different distribution in the target variable (see Supplementary Figure A.7.).

Overall, we see a clear benefit of using federated learning in patient-reported survey data when gathering local data into one server is impossible or if the data are scarce, as demonstrated in our subsampling experiment. As the sample size diminishes, the gap between local and centralized widens drastically, while the federated model keeps up with the centralized model. We also see that the local models are much more vulnerable to data diversity in international scenarios, while the federated model is robust to such data diversity.

Essentially, we demonstrated that for analyzing health survey data, one does not need to risk data privacy violations by centralizing patient-reported survey data on one server to secure model performance. Decentralized databases, according to the European Commission’s draft for the European Health Data Space (EHDS), can be materialized without affecting machine-learning-based prognostic model building for standardized distributed patient-reported survey data or health registries such as the *GLA: D*^®^ and SHARE registry. Whenever centralization of local data is not possible, federated learning could be applied for prognostic model building, preferably even if one data center has sufficient data for local model training, as these local models only reflect their region’s local population distributions. In contrast, federated learning across multiple regions trains more generalized models for the global population.

We further noticed that the local model originating from one of the five regions underperforms the other local models, which could be explained by the statistically significant difference in the distribution of the target variable (change in pain) and other variables (age, BMI, living situation) between region five and the other four regions. This illustrates that locally trained models may suffer heavily from data heterogeneity in real-world scenarios, where federated learning is less susceptible.

In our study, for the first time, we demonstrate the applicability of federated learning in real-life patient-reported survey data using federated linear regression, random forest regression, and random forest classification. The only requirement for federated learning is the availability of the same variables and target variables across all clients. Hence, whenever centralization of all isolated data is impossible or inconvenient, we suggest using federated learning to increase model performance by leveraging multi-center data, e.g., for developing personalized healthcare prediction models.

In our study, we were limited to two retrospective datasets, and as our goal is not to find the best model, clinical validation is not required. We merely show that existing models can be federated without a compromise in prediction accuracy. We used simple models like random forest and not neural networks, as it is shown that the random forest works better than neural networks in tabular data [53].

We added SMPC to the Linear regression model, which protects from direct disclosure attacks, data aggregation attacks, etc. We added DP to random forest, which protects from membership inference attacks, linkage attacks, etc.

In this work, we assume that participating data centers already have harmonized variables and a shared predictive goal, allowing us to focus on the technical aspects of federated learning. We are aware that there may exist a variety of real-world deployment barriers, such as infrastructure differences, network latency, varying data governance, and administrative and bureaucratic policies. Additionally, practical implementation in clinical or public health settings requires addressing non-technical barriers, including IT infrastructure, collaboration agreements, and user training. While all these factors need to be respected and should be addressed, the scope of this paper was to provide a technical solution when those barriers are solved in case-by-case scenarios.

## Conclusion

We evaluated the utility of federated learning technology and its applicability to health survey data for developing prediction models in healthcare research across nationwide and international scenarios. We demonstrated the applicability of federated machine learning in real-world datasets of self-reported health-survey-based data. In both the national and international datasets, we found the federated models comparable to those trained in a centralized fashion while performing better than models trained on local data. Henceforth, we showed that federated learning can be applied to handle the privacy vs. model accuracy dilemma in multi-centric, internationally distributed patient-reported healthcare datasets.

## Supplementary Information

Supplementary Material 1.

## Data Availability

All codes for data preprocessing, local and centralized models, evaluation, and details of repositories of the exact version of the feature cloud app can be accessed through our GitHub repository40. We have also added an HTML file for interactive plots where one can hover over the plots to see exact values in the same repository. For anonymized raw data access, one can apply to the co-leads of the *GLA: D* ^®^ Denmark’s registry directly (Prof. Roos and Prof. Skou), who will evaluate requests respecting the General Data Protection Regulation (GDPR). For SHARE data, one can apply it directly to their online portal[41].

## Abbreviations

R^2^: R-squared
RMSE: Root mean squared error
AUROC: Area under the receiver operating characteristic curve
OA: Osteoarthritis
GLA:D: Good life with osteoArthritis in Denmark
SHARE: Survey of health, ageing, and retirement in Europe
