# Supplementary material for: Privacy-preserving federated prediction of health outcomes using multi-center survey data
Source: BMC Med Res Methodol. 2026 Feb 4;26:46. doi: 10.1186/s12874-026-02785-5 (PMC12930927; doi:10.1186/s12874-026-02785-5)
Supplement: Supplementary file 1 — Supplementary Material 1. [file 12874_2026_2785_MOESM1_ESM.docx]

Supplementary Information

**Privacy-preserving federated prediction of pain intensity change based on multi-center survey data**

Supratim Das^a,b^, Mahdie Rafie^a,b^, Paula Kammer^a^, Søren T. Skou^c,d^, Dorte T. Grønne^c,d^, Ewa M. Roos^c^, André Hajek^b^, Hans-Helmut König^b^, Md Shihab Ullah^a^Niklas Probul^a^ Jan Baumbach^a,e^, Linda Baumbach^b^

*^a^Institute for Computational Systems Biology, University of Hamburg, Albert-Einstein-Ring 8-10, 22761, Hamburg, Germany*

*^b^Department of Health Economics and Health Services Research, University Medical Center Hamburg-Eppendorf, Martinistraße 52*

*Hamburg, 20246, Germany*

*^c^ Center for Muscle and Joint Health, Department of Sports Science and Clinical Biomechanics, University of Southern Denmark, Campusvej 55, 5230 Odense, Denmark*

*^d^ The Research and Implementation Unit PROgrez, Department of Physiotherapy and Occupational Therapy, Næstved-Slagelse-Ringsted Hospitals, Fælledvej 2C, 4200 Slagelse, Denmark*

*^e^ Computational Biomedicine Lab, Department of Mathematics and Computer Science, University of Southern Denmark, Odense, Denmark*

**Table of Contents:**

Supplementary Text: page 17-29

Statistical Test details: page 17-18

Supplementary Figures: page: 18-23

Supplementary Figure A.1 Federated Learning in Feature Cloud, Modus operandi: page: 18

Supplementary Figure A.2 Fair evaluation scheme: page: 19

Supplementary Figure A.3 Comparison of federated model with centralized and local models in SHARE: page: 20

Supplementary Figure A.4 Comparison of federated model with centralized and local models for 25% subsampled training data in GLA:D®: page: 21

Supplementary Figure A.5 Statistical test to evaluate data heterogeneity in GLA:D® data: (A) Posthoc Scheffe test: page: 22

Supplementary Figure A.6 Distribution of the target variable physical inactivity across 27 countries in SHARE data: page: 23

Supplementary Tables: page: 24-29

Supplementary Table B GLA:D® data variable description: page: 24- 26

Supplementary Table B.1 Sample size distribution across 27 countries in SHARE data: page: 27

Supplementary Table B.1 SHARE data variable description: page: 27- 29

# Supplementary Text:

Statistical Tests details: For the algorithm we will denote results of average of Locals as L , centralised as C, and federated model as F.

Algorith for statistical test is as follows:

|if for the metric higher is better : (e.g. R-squared, AUROC, Accuracy)

| |calculate the p-values for normality of L,C,F using Shapiro’s test.

| |calculate the p-values for test equality of distribution for L vs C, L vs F, and C vs F using | Bartlett's test.

| |if the Shapiro test’s p-value for L, C, and p-value for equality of distribution of L vs C is >0.05:

| | |calculate students' t-test, alternate hypothesis C>L

| |else: calculate Welch’s t-test, alternate hypothesis C>L

|

| |if the Shapiro test’s p-value for L, F, and p-value for equality of distribution of L vs F is >0.05:

| | |calculate students' t-test, alternate hypothesis F>L

| |else: calculate Welch’s t-test, alternate hypothesis F>L

|

| |if the Shapiro test’s p-value for C, F, and p-value for equality of distribution of C vs F is >0.05:

| | |calculate students' t-test, alternate hypothesis C>F

| |else: calculate Welch’s t-test, alternate hypothesis C>F

|

|if for the metric lower is better : (e.g. RMSE)

| |calculate the p-values for normality of L,C,F using Shapiro’s test.

| |calculate the p-values for test equality of distribution for L vs C, L vs F and C vs F using | Bartlett's test.

| |if the Shapiro test’s p-value for L, C, and p-value for equality of distribution of L vs C is >0.05:

| | |calculate students' t-test, alternate hypothesis C<L

| |else: calculate Welch’s t-test, alternate hypothesis C<L

|

| |if the Shapiro test’s p-value for L, F, and p-value for equality of distribution of L vs F is >0.05:

| | |calculate students' t-test, alternate hypothesis F<L

| |else: calculate Welch’s t-test, alternate hypothesis F<L

|

| |if the Shapiro test’s p-value for C, F, and p-value for equality of distribution of C vs F is >0.05:

| | |calculate students' t-test, alternate hypothesis C<F

| |else: calculate Welch’s t-test, alternate hypothesis C<F

# Supplementary Figures


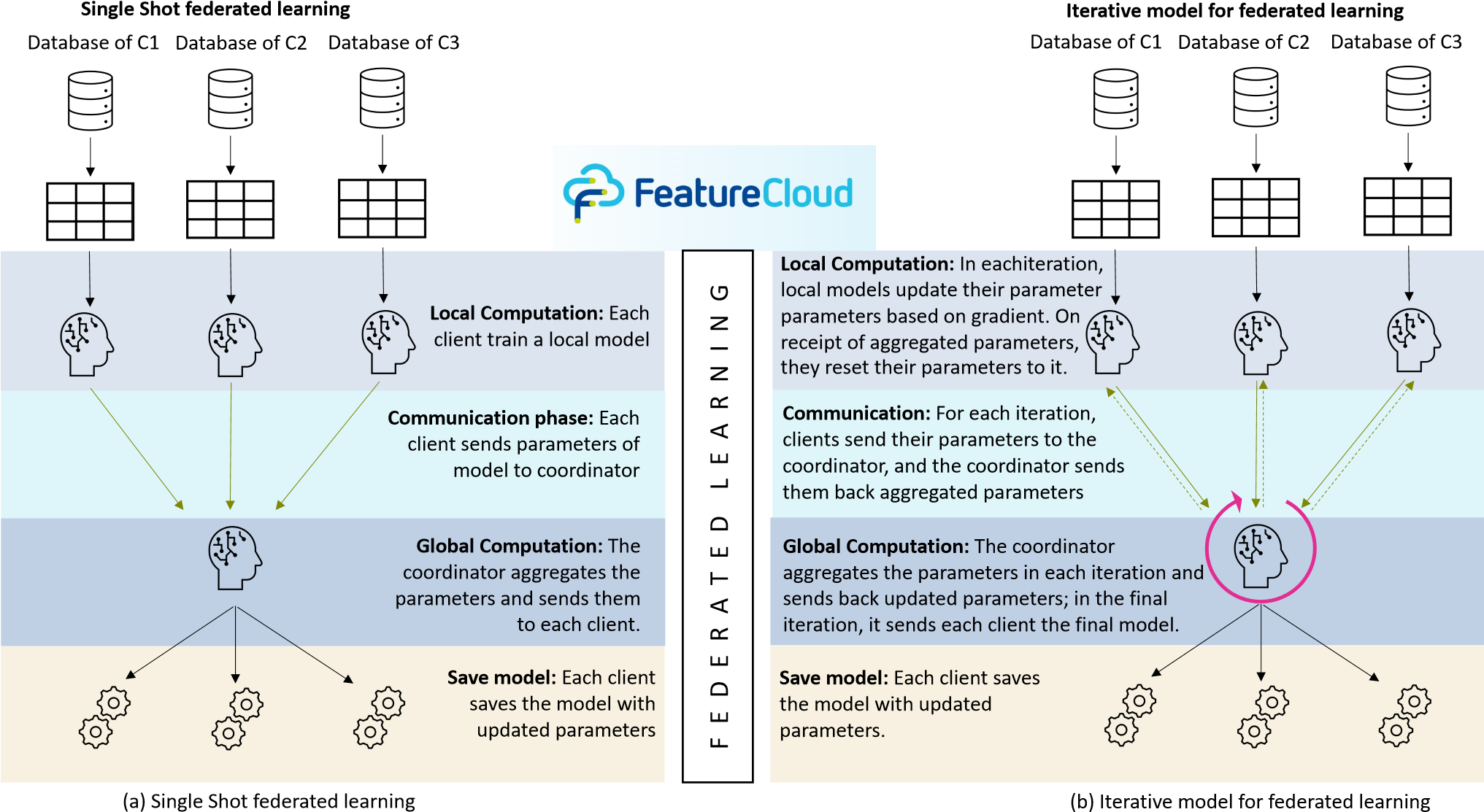


Supplementary Figure A.1: **Federated Learning in Feature Cloud, Modus operandi**: Federated learning of any centralized model can be achieved in two ways. One may use one-time communication of local models with the aggregator and update the aggregated parameters. Simpler models can be built in this manner. Another way is to have multiple such communication rounds (iterations) until convergence.



Supplementary Figure A.2: **Fair evaluation scheme**: For a fair comparison of local models with centralized and federated models and to simulate a clinically relevant scenario, we designed the same test set for all in each fold of 5-fold cross-validation. 20% of each local data was taken out (in pink) and combined to create this global identical test set. For training, each region used the rest of their data (in blue) or federated and local training, while for centralized training, a set combination of all the training data was used.


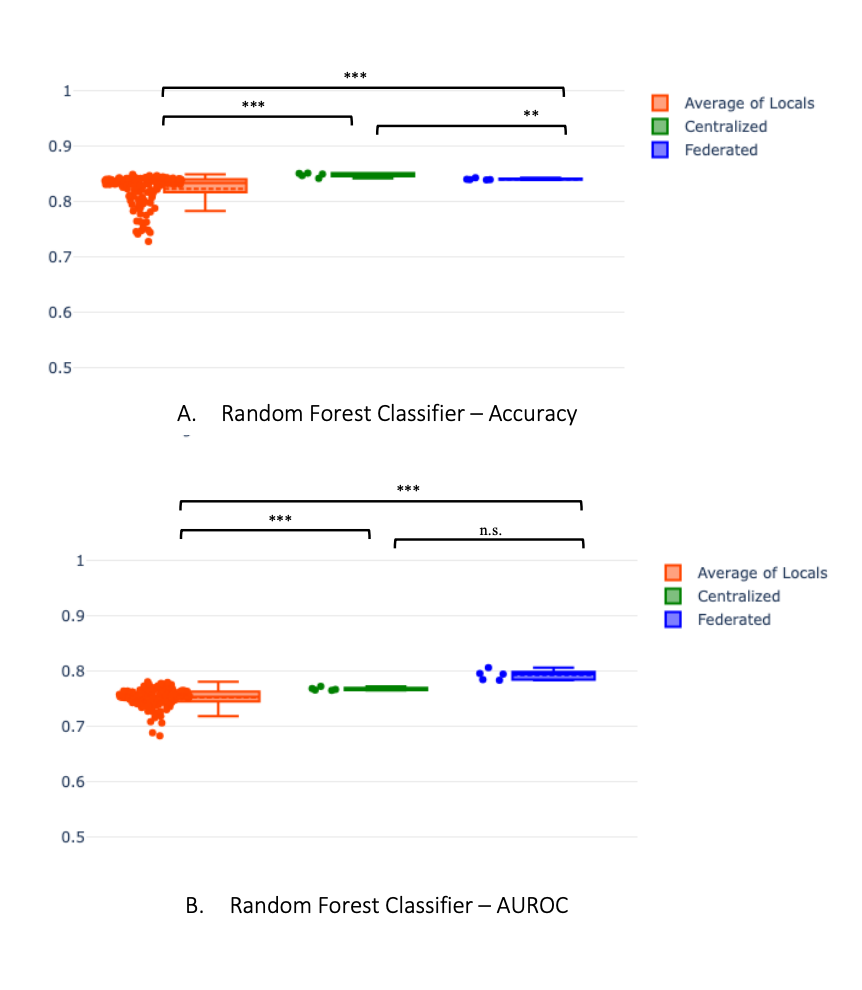


Supplementary Figure A.3. **Comparison of federated model with centralized and local models in SHARE**: In **A.**, the random forest classifier accuracy in the centralized model is higher than that of the federated, and certain local models seem to be as good as the federated model. However, the mean (dotted line) for the average of locals is much lower because most of the local models are underperforming. The federated learning has much less variance than the local model. In real life, finding a local model that performs good in centralized data is not possible either. In **B.**, the random forest classifier AUROC in the federated model is even better than the centralized model. This suggests that federated learning is more robust. The dotted lines in the box plots represent the mean, while the solid lines represent the median. The stars represent the magnitude of the significance of the tested hypothesis in the following convention *** : p<0·001, **: p<0·01, * : p<0·05, n.s. : p>0·05. We tested the following hypotheses: 1. if the centralized model is better than the average of local ones, 2. if the federated is better than the average of local ones, and 3. if the centralized is better than the federated.


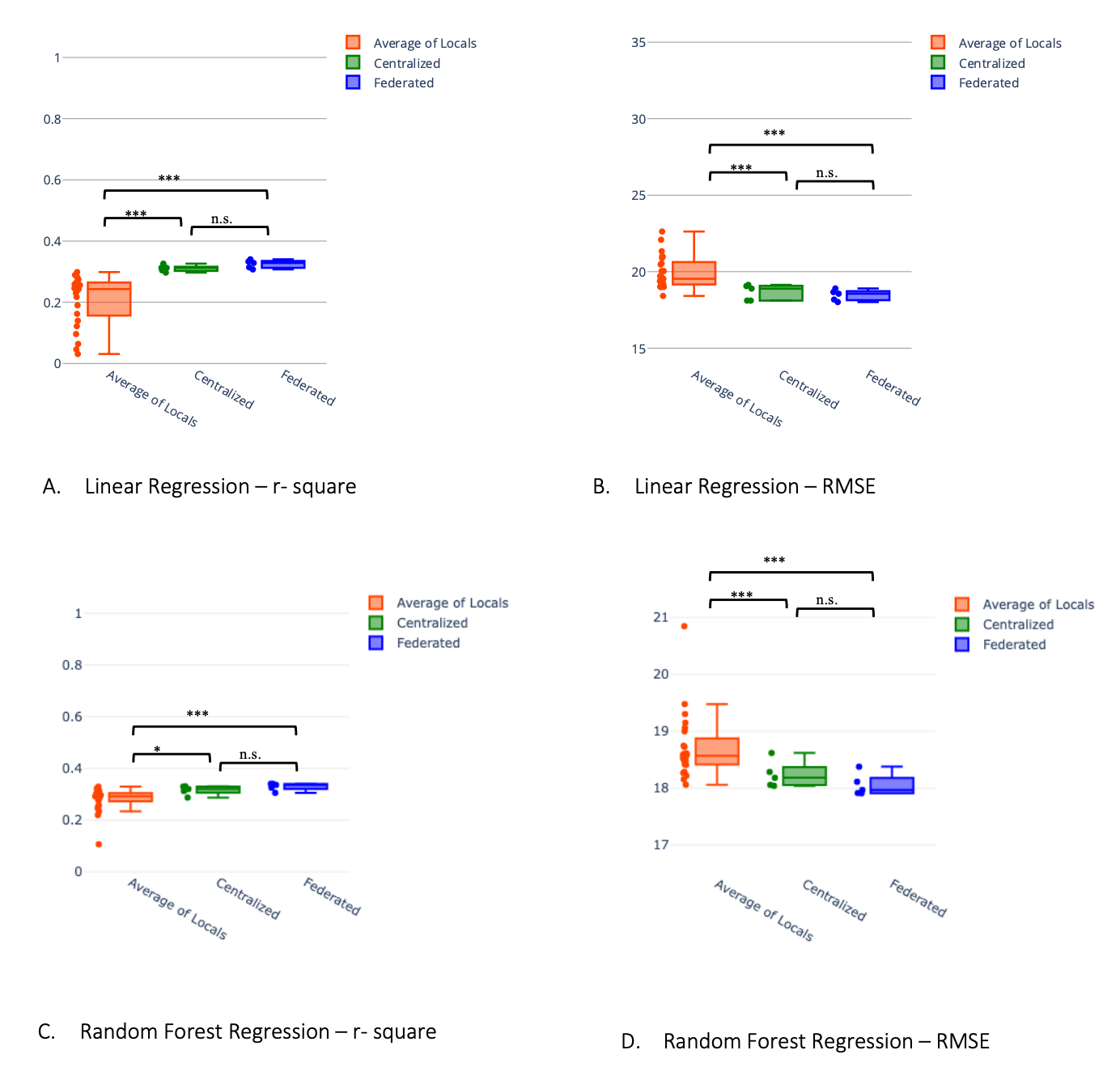


Supplementary Figure A.4. **Comparison of federated model with centralized and local models for 25% subsampled training data in *GLA:D*®**: For both **A.** R-square of linear regression (the higher, the better) and **B.** For the RMSE of linear regression (lower, better), we see that federated and centralized models perform better than the local models. Similarly, for both **C.** R-square of random forest regression (higher the better) and **D.** RMSE of random forest regression (lower the better), we see that the federated model outperforms both local and central models. The dotted lines in the box plots represent the mean, while the solid lines represent the median. The stars represent the magnitude of the significance of the tested hypothesis in the following convention *** : p<0·001, **: p<0·01, * : p<0·05, n.s. : p>0·05. We tested the following hypotheses: 1. if the centralized model is better than the average of local ones, 2. if the federated is better than the average of local ones, and 3. if the centralized is better than the federated.


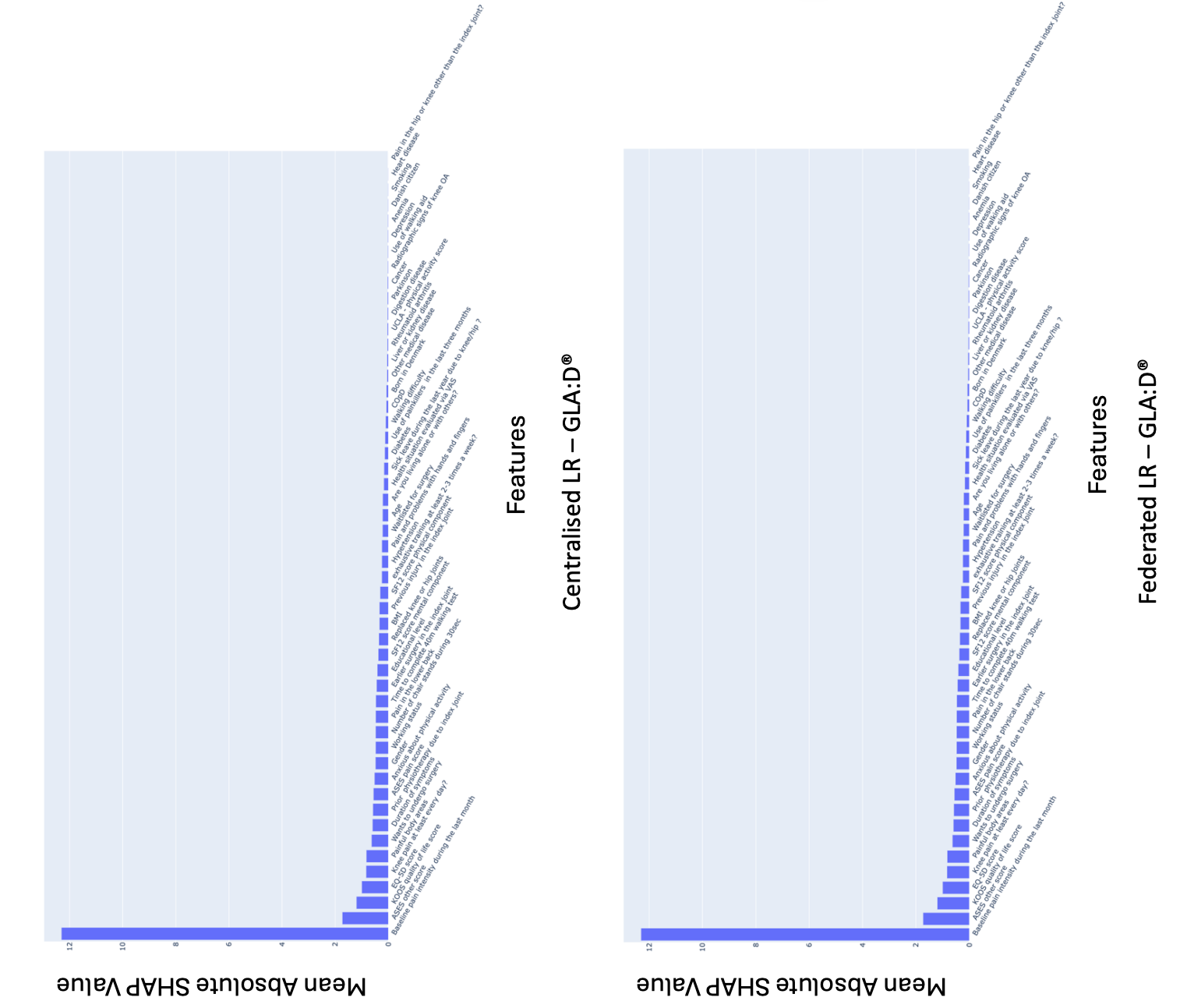


Supplementary Figure A.5.A. **Comparison of SHAP-based feature importance, federated centralized version of Linear regression.**


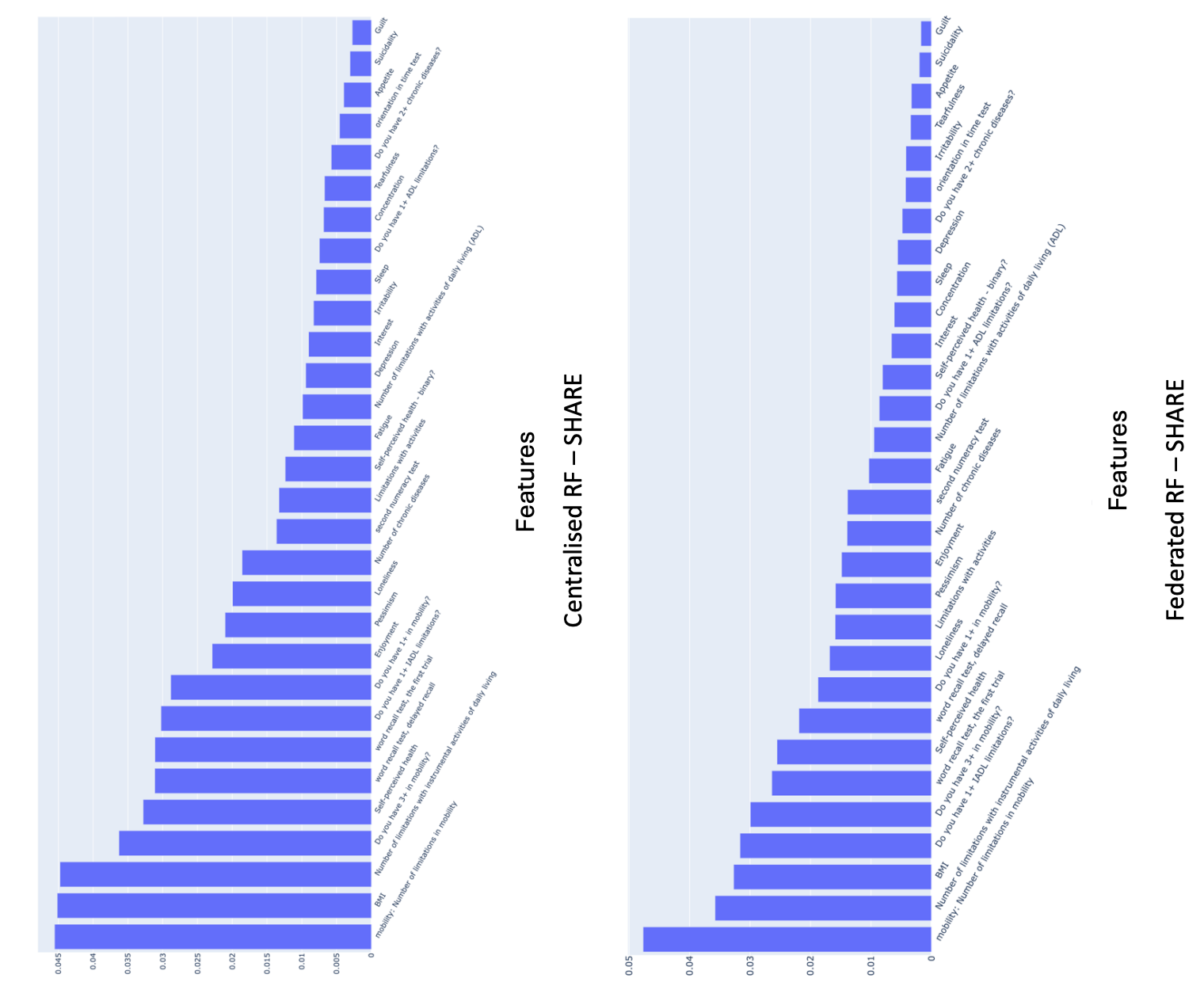


Supplementary Figure A.5.B. **Comparison of SHAP-based feature importance federated centralized version Random forest classification.**


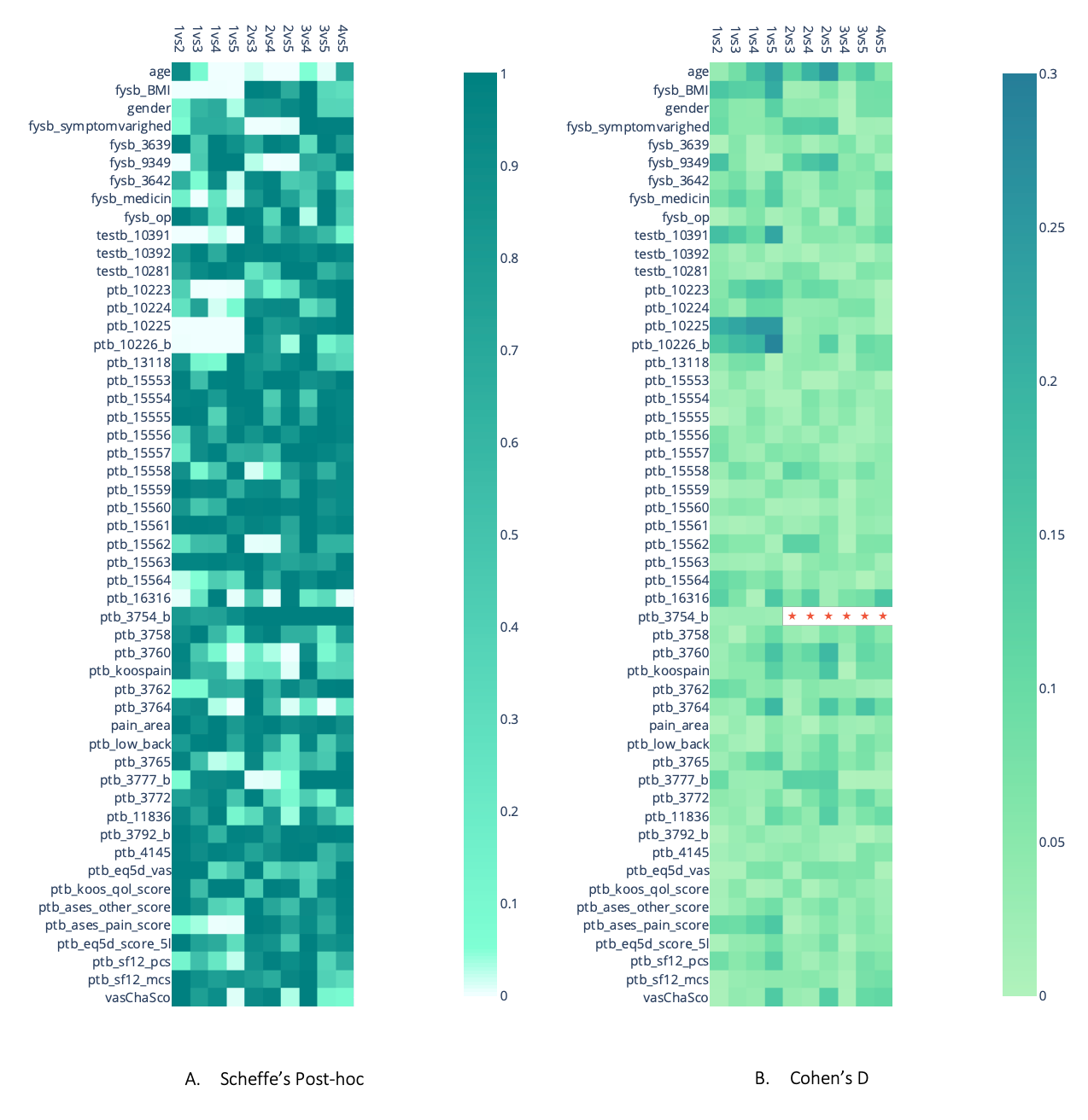


Supplementary Figure A.6: **Statistical test to evaluate data heterogeneity in *GLA:D*® data**: **A. Posthoc Scheffe test:** We used post hoc Scheffe to test pairwise if two regions’ data originates from different distributions. The comparison of two regions, X and Y, is denoted as “XvsY.” We did this for all 51 variables and the target variable. A light color suggests statistical significance, while green suggests no significance.

**B. Cohen’s-D:** This shows the Cohen’s -D effect size of each statistical test; the darker the color, the higher the effect size. Red stars suggest missing values. Although the means of the distribution are close, we find statistically significant differences among regions in some variables, including the target variable.


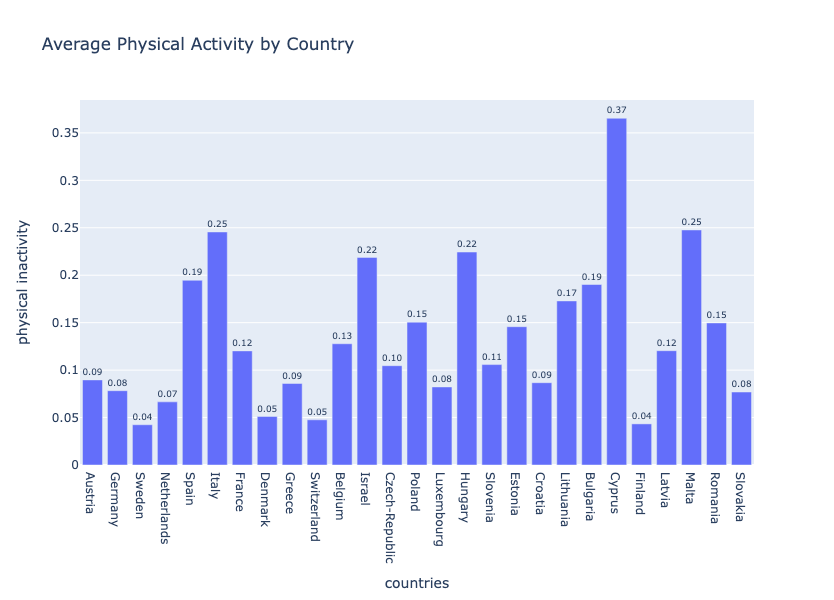


Supplementary Figure A.7: **Distribution of the target variable physical inactivity across 27 countries in SHARE data:** In the y-axis, we plot the average level of physical inactivity in each country.

# Supplementary Tables

# Supplementary Table B.1: GLA:D® data variable description. (Abbreviations used: OA: Osteoarthritis, NSAID: non-steroidal anti-inflammatory drugs, VAS: visual analog scale, KOOS: Knee Injury and Osteoarthritis Outcome Score, ASES: Arthritis Self Efficacy Scale, EQ-5D: EuroQol- 5 dimensions.SF-12” 12-Item Short Form Survey.)

| **Variable ID** | **Variable Name** | **Mean, standard deviation, (SD) or number of cases**  **(total n =9648)** |
| --- | --- | --- |
| age | 1. Age | Mean:64·93  SD:9·40  Min:23  Max:94 |
| fysb_−_BMI | 2. BMI | Mean:28·60  SD:5·27  Min:15·23  Max:70·03 |
| gender | 3. Gender | Men:2725  Women:6923 |
| fysb_−_symptomvarighed | 4. Duration of symptoms | Mean:43·91  SD:67·76  Min:0·0  Max:756·0 |
| fysb_−_3639 | 5. Waitlisted for surgery | Yes:166  No:9482 |
| fysb_−_9349 | 6. Radiographic signs of knee OA | Yes:7639  No:373  Unknown:1636 |
| fysb_−_3642 | 7. Prior physiotherapy due to index joint | Yes:3241  No:6407 |
| fysb_−_medicin | 8. Use of painkillers (paracetamol/ NSAID/ opioids/ codeine) in the last three months | Yes:6123  No:3525 |
| fysb_−_op | 9. Earlier surgery in the index joint | Yes:2681  No:6967 |
| testb_−_10391 | 10. Time to complete 40m walking test | Mean:28·18  SD:7·48  Min:10·0  Max:234·91 |
| testb_−_10392 | 11. Use of walking aid | Yes:159  No:9489 |
| testb_−_10281 | 12. Number of chair stands during 30sec | Mean:11·95  SD:3·67  Min:0·0  Max:33·0 |
| ptb_−_10223 | 13. Born in Denmark | Yes:9287  No:361 |
| ptb_−_10224 | 14. Danish citizen | Yes:9490  No:158 |
| ptb_−_10225 | 15. Are you living alone or with others? | Living aolne:2347  Living with others:7301 |
| ptb_−_10226_−_b | 16. Educational level  (Do you have an education higher than secondary education? ) | Yes:6863  No:2785 |
| ptb_−_13118 | 17. Smoking | Yes:755  No:8893 |
| ptb_−_15553 | 18. Hypertension | Yes:3652  No:5996 |
| ptb_−_15554 | 19. Heart disease | Yes:699  No:8949 |
| ptb_−_15555 | 20. Lung disease e.g. COpD | Yes:552  No:9096 |
| ptb_−_15556 | 21. Diabetes | Yes:675  No:8973 |
| ptb_−_15557 | 22. Digestion disease | Yes:445  No:9203 |
| ptb_−_15558 | 23. Liver or kidney disease | Yes:113  No:9535 |
| ptb_−_15559 | 24. Blood disease e.g. Anemia | Yes:107  No:9541 |
| ptb_−_15560 | 25. Cancer | Yes:254  No:9394 |
| ptb_−_15561 | 26. Depression | Yes:365  No:9283 |
| ptb_−_15562 | 27. Rheumatoid arthritis | Yes:468  No:9180 |
| ptb_−_15563 | 28. Neurological disease e.g. Parkinson | Yes:457  No:9191 |
| ptb_−_15564 | 29. Other medical disease | Yes:1743  No:7905 |
| ptb_−_16316 | 30. Previous injury in the index joint serious enough to consult a medical doctor | Yes:4951  No:4697 |
| ptb_−_3754_−_b | 31. Pain in the hip or knee other than the index joint? | Yes:1  No:9647 |
| ptb_−_3758 | 32. Walking difficulty | Yes:7397  No:2251 |
| ptb_−_3760 | 33. Pain and problems with hands and fingers | Yes:3229  No:6419 |
| ptb_−_koospain | 34. Knee pain at least every day? | Yes:7782  No:1866 |
| ptb_−_3762 | 35. Anxious about physical activity | Yes:1429  No:8219 |
| ptb_−_3764 | 36. Baseline pain intensity during the last month (VAS scale 0-100, no pain to worst pain | Mean:46·76  SD:21·68  Min:0·0  Max:100·0 |
| pain_−_area | 37. Painful body areas (collected via pain drawing) | Mean:3·45  SD:3·26  Min:0·0  Max:40·0 |
| ptb_−_low_−_back | 38. Pain in the lower back (collected via pain drawing) | Yes:2334  No:7314 |
| ptb_−_3765 | 39. Wants to undergo surgery | Yes:1037  No:8611 |
| ptb_−_3777_−_b | 40. Working status  Are you working/ studying? | Working/Studing:2875  No:6773 |
| ptb_−_3772 | 41. Sick leave during the last year because of knee/hip during last year? | Yes:1085  No:8563 |
| ptb_−_11836 | 42. Replaced knee or hip joints | Yes:877  No:8771 |
| ptb_−_3792_−_b | 43. Frequency of training until exhaustion at least 2-3 times a week? | Yes:4913  No:4735 |
| ptb_−_4145 | 44. UCLA - physical activity score  (from 0 : 10 worst to best) | Mean:5·76  SD:1·79  Min:1·0  Max:10·0 |
| ptb_−_eq5d_−_vas | 45. Health situation evaluated via VAS  (from 0 : 100, worst to best) | Mean:70·43  SD:18·41  Min:0·0  Max:100·0 |
| ptb_−_koos_−_qol_−_score | 46. KOOS quality of life score  (from 0 : 100, worst to best) | Mean:45·79  SD:15·06  Min:0·0  Max:100·0 |
| ptb_−_ases_−_pain_−_score | 47. ASES pain score  (from 10 : 100, worst to best) | Mean:67·63  SD:19·26  Min:10·0  Max:100·0 |
| ptb_−_ases_−_other_−_score | 48. ASES other score (from 10 : 100, worst to best) | Mean:71·54  SD:17·20  Min:10·0  Max:100·0 |
| ptb_−_eq5d_−_score_−_5l | 49. EQ-5D score  From -0.624 : 1, worst to best) | Mean:0·78  SD:0·18  Min:-0·37  Max:1·0 |
| ptb_−_sf12_−_pcs | 50. SF12 score physical component  (from 0 : 100, worst to best) | Mean:38·27  SD:8·85  Min:11·03  Max:65·95 |
| ptb_−_sf12_−_mcs | 51. SF12 score mental component  (from 0 : 100, worst to best) | Mean:53·03  SD:9·43  Min:11·62  Max:70·99 |
| vasChaSco | 52. Improvement in VAS pain score from baseline to 3 months follow-up score  -100 : 100, from pain got worse to pain got better | Mean:14·23  SD:22·48  Min:-87·0  Max:99·0 |

Supplementary Table B.2: **Sample size distribution across 27 countries in SHARE data.**

| **Country** | **Patients** | **Country** | **Patients** | **Country** | **Patients** |
| --- | --- | --- | --- | --- | --- |
| Austria | 1483 | Switzerland | 1869 | Croatia | 1153 |
| Germany | 2797 | Belgium | 1941 | Lithuania | 1411 |
| Sweden | 2286 | Israel | 809 | Bulgaria | 899 |
| Netherlands | 1879 | Czech-Republic | 2599 | Cyprus | 509 |
| Spain | 1952 | Poland | 1986 | Finland | 1108 |
| Italy | 2044 | Luxembourg | 863 | Latvia | 772 |
| France | 2403 | Hungary | 753 | Malta | 763 |
| Denmark | 2130 | Slovenia | 2353 | Romania | 1269 |
| Greece | 2933 | Estonia | 2861 | Slovakia | 988 |

Supplementary Table B.3: **SHARE data variable description.(** sphus = Self-Perceived Health US version, Abbreviations used: ADL = Activities of daily living, IADL = Limitations with instrumental activities of daily living)

| **Variable id** | **Variable name** | **Mean, standard deviation, (SD) or number of cases**  **(total n =44813)** |
| --- | --- | --- |
| bmi | 1. BMI | Mean: 27·17  SD: 4·73  Min: 12·46  Max: 97·78  Don’t know/ Refusal to answer/suspected wrong: 1205 |
| sphus | 2. Self-perceived health (US version)  (Based on SF-36 questionnaire )  (from 1 : 5, excellent to poor) | Mean: 3·19  SD: 1·0  Min: 1  Max: 5  Don’t know/ Refusal to answer: 12 |
| sphus2 | 3. Do you have less than very good health in self-perceived health (sphus)? | Yes: 34689  No: 10112  Don’t know/ Refusal to answer: 12 |
| chronic2w8 | 4. Do you have 2+ chronic diseases? | Yes: 23680  No: 21102  Don’t know/ Refusal to answer: 31 |
| chronicw8c | 5. Number of chronic diseases | Mean: 1·90  SD: 1·60  Min: 0  Max: 14  Don’t know/ Refusal to answer: 31 |
| numeracy2 | 6. Score of second numeracy test  (from 0 : 5, bad to good) | Mean: 4·11  SD: 1·41  Min: 0  Max: 5  Don’t know/ Refusal to answer: 0 |
| orienti | 7. Score of orientation in time test  (from 0 : 4, bad to good) | Mean: 3·83  SD: 0·53  Min: 0  Max: 4  Don’t know/ Refusal to answer: 0 |
| cf008tot | 8. Ten words list learning the first trial | Mean: 5.27  SD: 1.75  Min: 0  Max:10 |
| cf016tot | 9. Ten words list learning delayed recall | Mean: 3·87  SD: 2·14  Min: 0  Max: 10 |
| euro1 | 10. Depression  In the last month, have you been sad or depressed?  (part of EURO-D measure of depressive Symptoms) | Yes: 17775  No: 26985  Don’t know/ Refusal to answer: 53 |
| euro2 | 11. Pessimism  What are your hopes for the future?  (part of EURO-D measure of depressive Symptoms) | Any hopes mentioned: 7895  No such mentioned: 36815  Don’t know/ Refusal to answer: 103 |
| euro3 | 12. Suicidality  In the last month, have you felt that you would rather be dead?  (part of EURO-D measure of depressive Symptoms) | Any such mention: 2634  No such mention: 42093  Don’t know/ Refusal to answer: 86 |
| euro4 | 13. Guilt  Do you tend to blame yourself or feel guilty about anything?  (part of EURO-D measure of depressive Symptoms) | Obvious mention of guilt / self-blame: 3370  No: 41366  Don’t know/ Refusal to answer: 77 |
| euro5 | 14. Sleep  Have you had trouble sleeping recently?  (part of EURO-D measure of depressive Symptoms) | Yes: 16362  No: 28428  Don’t know/ Refusal to answer: 23 |
| euro6 | 15. Interest  In the last month, what is your interest in things?  (part of EURO-D measure of depressive Symptoms) | Less interest than normal: 4771  No mention of the loss of interest: 40007  Don’t know/ Refusal to answer: 35 |
| euro7 | 16. Irritability  Have you been irritable recently?  (part of EURO-D measure of depressive Symptoms) | Yes: 33211  No: 11558  Don’t know/ Refusal to answer: 44 |
| euro8 | 17. Appetite  What has your appetite been like?  (part of EURO-D measure of depressive Symptoms) | Diminution in desire for food: 4196  No diminution: 40606  Don’t know/ Refusal to answer: 11 |
| euro9 | 18. Fatigue  In the last month, have you had too little energy to do things you wanted to do?  (part of EURO-D measure of depressive Symptoms) | Yes: 15748  No: 29020  Don’t know/ Refusal to answer: 45 |
| euro10 | 19. Concentration  How is your concentration?  (part of EURO-D measure of depressive Symptoms) | Difficulty in concentration: 7574  No difficulty: 37215  Don’t know/ Refusal to answer: 24 |
| euro11 | 20. Enjoyment  What have you enjoyed doing recently?  (part of EURO-D measure of depressive Symptoms) | Fails to mention any: 6027  Mentioned any activity: 38722  Don’t know/ Refusal to answer: 64 |
| euro12 | 21. Tearfulness  In the last month, have you cried at all?  (part of EURO-D measure of depressive Symptoms) | Yes: 10617  No: 34155  Don’t know/ Refusal to answer: 41 |
| loneliness | 22. Loneliness (short version of R-UCLA Loneliness Scale)  (from 3: 9, not lonely to very lonely) | Mean: 3·96  SD: 1·41  Min: 3  Max: 9  Don’t know/ Refusal to answer: 0 |
| gali | 23. Limitations with activities | Yes: 21660  No: 23137  Don’t know/ Refusal to answer: 16 |
| mobility | 24. Number of limitations in mobility, arm function, and fine motor limitations | Mean: 1·71  SD: 2·37  Min: 0  Max: 10  Don’t know/ Refusal to answer: 14 |
| mobilit2 | 25. Do you have 1+ mobility, arm function, and fine motor limitations? | Yes: 23069  No: 21730  Don’t know/ Refusal to answer: 14 |
| mobilit3 | 26. Do you have 3+ mobility, arm function, and fine motor limitations? | Yes: 11983  No: 32816  Don’t know/ Refusal to answer: 14 |
| adl | 27. Number of limitations with activities of daily living (ADL) | Mean: 0·22  SD: 0·81  Min: 0  Max: 6  Don’t know/ Refusal to answer: 20 |
| adl2 | 28. Do you have 1+ ADL limitations? | Yes: 4910  No: 39883  Don’t know/ Refusal to answer: 20 |
| iadl | 29. Number of limitations with instrumental activities of daily living | Mean: 0·50  SD: 1·42  Min: 0  Max: 9  Don’t know/ Refusal to answer: 20 |
| iadl2 | 30. Do you have 1+ IADL limitations? | Yes: 8238  No: 36555  Don’t know/ Refusal to answer: 20 |
| inact | 31. Calculated physical inactivity | Yes: 8238  No: 36575 |

Supplementary Table B.4: **ANOVA on GLA:D® data to check difference in mean of distribution across 5 regions. (**E: exponent of 10, i.e. 2.5E-05 = 2.5*$10^{-5}$)

| *Variable* | *Variable Name* | *F-statistic* | *p-value* |
| --- | --- | --- | --- |
| *age* | *Age* | *18.2175479* | *6.40E-15* |
| *fysb_BMI* | *BMI* | *10.8331694* | *9.23E-09* |
| *gender* | *Gender* | *3.43976432* | *0.008135* |
| *fysb_symptomvarighed* | *Duration of symptoms* | *6.65694491* | *2.40E-05* |
| *fysb_3639* | *Waitlisted for surgery* | *1.70461333* | *0.145895* |
| *fysb_9349* | *Radiographic signs of knee OA* | *7.84198531* | *2.63E-06* |
| *fysb_3642* | *Prior physiotherapy due to the index joint* | *4.02649326* | *0.002895* |
| *fysb_medicin* | *Use of painkillers (paracetamol/ NSAID/ opioids/ codeine) in the last three months* | *6.03370541* | *7.60E-05* |
| *fysb_op* | *Earlier surgery in the index joint* | *2.97885918* | *0.018039* |
| *testb_10391* | *Time to complete the 40m walking test* | *10.7591262* | *1.06E-08* |
| *testb_10392* | *Use of a walking aid* | *0.74860983* | *0.558782* |
| *testb_10281* | *Number of chair stands during 30sec* | *1.79763612* | *0.12625* |
| *ptb_10223* | *Born in Denmark* | *9.63564043* | *8.96E-08* |
| *ptb_10224* | *Danish citizen* | *4.16979769* | *0.002244* |
| *ptb_10225* | *Are you living alone or with others?* | *26.4016346* | *8.29E-22* |
| *ptb_10226_b* | *Educational level* | *17.9698448* | *1.03E-14* |
| *ptb_13118* | *Smoking* | *3.16989197* | *0.012992* |
| *ptb_15553* | *Hypertension* | *0.85099207* | *0.492668* |
| *ptb_15554* | *Heart disease* | *1.32156998* | *0.259249* |
| *ptb_15555* | *COpD* | *1.2530208* | *0.28614* |
| *ptb_15556* | *Diabetes* | *1.27344489* | *0.277891* |
| *ptb_15557* | *Digestion disease* | *1.73163315* | *0.139918* |
| *ptb_15558* | *Liver or kidney disease* | *4.13983733* | *0.002367* |
| *ptb_15559* | *Anemia* | *0.90806625* | *0.458099* |
| *ptb_15560* | *Cancer* | *1.18285457* | *0.316056* |
| *ptb_15561* | *Depression* | *0.58638701* | *0.672497* |
| *ptb_15562* | *Rheumatoid arthritis* | *4.88753321* | *0.000618* |
| *ptb_15563* | *Parkinson* | *0.95230401* | *0.432492* |
| *ptb_15564* | *Other medical diseases* | *3.75227503* | *0.004703* |
| *ptb_16316* | *Previous injury in the index joint was serious enough to consult a medical doctor* | *7.91763701* | *2.29E-06* |
| *ptb_3754_b* | *Pain in the hip or knee other than the index joint?* | *0.74867974* | *0.558735* |
| *ptb_3758* | *Walking difficulty* | *2.36619591* | *0.050552* |
| *ptb_3760* | *Pain and problems with hands and fingers* | *7.13837755* | *9.81E-06* |
| *ptb_koospain* | *Knee pain at least every day?* | *4.43229652* | *0.001403* |
| *ptb_3762* | *Anxious about physical activity* | *3.14948506* | *0.013457* |
| *ptb_3764* | *Baseline pain intensity during the last month (VAS scale 0-100, no pain to worst pain* | *7.50973853* | *4.91E-06* |
| *pain_area* | *Painful body areas (collected via pain drawing)* | *0.65143019* | *0.625825* |
| *ptb_low_back* | *Pain in the lower back (collected via pain drawing)* | *1.62024433* | *0.166088* |
| *ptb_3765* | *Wants to undergo surgery* | *4.3532083* | *0.001617* |
| *ptb_3777_b* | *Working status* | *4.71569144* | *0.000843* |
| *ptb_3772* | *Sick leave during the last year because of the knee/hip during last year?* | *2.95285725* | *0.018858* |
| *ptb_11836* | *Replaced knee or hip joints* | *3.1156198* | *0.014265* |
| *ptb_3792_b* | *Frequency of training until exhaustion at least 2-3 times a week?* | *0.80898115* | *0.519181* |
| *ptb_4145* | *UCLA - physical activity score* | *1.04183478* | *0.383893* |
| *ptb_eq5d_vas* | *Health situation evaluated via VAS* | *2.36863786* | *0.050349* |
| *ptb_koos_qol_score* | *KOOS quality of life score* | *1.47849437* | *0.205757* |
| *ptb_ases_other_score* | *ASES pain score* | *1.86403038* | *0.113754* |
| *ptb_ases_pain_score* | *ASES other score (from 10: 100, worst to best)* | *6.08939952* | *6.86E-05* |
| *ptb_eq5d_score_5l* | *EQ-5D score* | *1.64437956* | *0.160067* |
| *ptb_sf12_pcs* | *SF12 score physical component* | *3.71700216* | *0.005005* |
| *ptb_sf12_mcs* | *SF12 score mental component* | *1.74264059* | *0.137548* |
| *vasChaSco* | *Improvement in VAS pain score from baseline to 3 months follow-up score* | *3.77650065* | *0.004507* |
